# Ethnicity-specific epigenetic variation in naïve CD4+ T cells and the susceptibility to autoimmunity

**DOI:** 10.1186/s13072-015-0037-1

**Published:** 2015-11-24

**Authors:** Patrick Coit, Mikhail Ognenovski, Elizabeth Gensterblum, Kathleen Maksimowicz-McKinnon, Jonathan D. Wren, Amr H. Sawalha

**Affiliations:** Division of Rheumatology, University of Michigan, 5520 MSRB-1, SPC 5680, 1150 W. Medical Center Drive, Ann Arbor, MI 48109 USA; Division of Rheumatology, Henry Ford Health System, 3031 W Grand Blvd, Detroit, MI 48202 USA; Arthritis and Clinical Immunology Program, Oklahoma Medical Research Foundation, 825 NE 13th St, MS 53, Oklahoma City, OK 73104 USA; Department of Biochemistry and Molecular Biology, The University of Oklahoma Health Sciences Center, 1100 N Lindsay Ave, Oklahoma City, OK 73104 USA; Center for Computational Medicine and Bioinformatics, University of Michigan, 100 Washtenaw Ave, #2017, Ann Arbor, MI 48109 USA

**Keywords:** Epigenetic, Autoimmunity, Lupus, Ethnicity specific, Genetic, T cell

## Abstract

**Background:**

Genetic and epigenetic variability contributes to the susceptibility and pathogenesis of autoimmune diseases. T cells play an important role in several autoimmune conditions, including lupus, which is more common and more severe in people of African descent. To investigate inherent epigenetic differences in T cells between ethnicities, we characterized genome-wide DNA methylation patterns in naïve CD4+ T cells in healthy African-Americans and European-Americans, and then confirmed our findings in lupus patients.

**Results:**

Impressive ethnicity-specific clustering of DNA methylation profiling in naïve CD4+ T cells was revealed. Hypomethylated loci in healthy African-Americans were significantly enriched in pro-apoptotic and pro-inflammatory genes. We also found hypomethylated genes in African-Americans to be disproportionately related to autoimmune diseases including lupus. We then confirmed that these genes, such as *IL32*, *CD226*, *CDKN1A*, and *PTPRN2* were similarly hypomethylated in lupus patients of African-American compared to European-American descent. Using patch DNA methylation and luciferase reporter constructs, we showed that methylation of the *IL32* promoter region reduces gene expression in vitro. Importantly, bisulfite DNA sequencing demonstrated that *cis*-acting genetic variants within and directly disrupting CpG sites account for some ethnicity-specific variability in DNA methylation.

**Conclusion:**

Ethnicity-specific inherited epigenetic susceptibility loci in CD4+ T cells provide clues to explain differences in the susceptibility to autoimmunity and possibly other T cell-related diseases between populations.

**Electronic supplementary material:**

The online version of this article (doi:10.1186/s13072-015-0037-1) contains supplementary material, which is available to authorized users.

## Background

DNA methylation is part of the epigenetic regulatory system in the cell and serves as an intermediary between an individual’s environment and their genetic background. One classic example is monozygotic twins; they share a nearly identical genotype, but display increased differences in DNA methylation and histone acetylation as they age [1]. Differences in DNA methylation also contribute to the phenotypic variation present between individuals and ethnicities. Epigenetic differences between ethnicities are an important consideration when investigating autoimmune disorders with a pattern of heterogeneous risk. Systemic lupus erythematosus (SLE, or lupus) and scleroderma both show a preponderance of risk and younger age of onset among African-Americans compared to European-Americans, while type-1 diabetes and multiple sclerosis have higher incidence rates among European-Americans [2]. Ethnicity can also be associated with disease severity and outcome. In a survey of SLE patients in southeastern Michigan, there was a 2.2-fold increase in renal involvement and 3.4-fold increase in end-stage renal disease in African-Americans compared to European-Americans [3].

Previous studies of the DNA methylation patterns in different ethnicities revealed that peripheral blood cells from non-Hispanic African-Americans display a global decrease in methylation of the retrotransposon LINE-1 compared to European-Americans [4]. DNA methylation differences seen in individuals of African-American, European-American, and Han Chinese ancestry were associated with genes related to xenobiotic metabolism and immune system function, among others [5]. These examples of epigenetic variation between ethnicities may provide answers as to what contribution inherent epigenetic differences make to the development and progression of autoimmune diseases.

DNA methylation changes in CD4+ T cells play an important role in the pathogenesis and clinical presentation of SLE, and these changes can be identified in naïve CD4+ T cells prior to T cell activation [6, 7]. Because SLE is more common and more severe in African-Americans compared to European-Americans, we hypothesized that inherent T cell DNA methylation differences might exist in African-Americans compared to European-Americans. To address this hypothesis, we evaluated the DNA methylome of naïve CD4+ T cells in healthy African-American and European-American populations, and then confirmed our findings in SLE patients.

## Results

### DNA methylation differences in naïve CD4+ T cells between African-Americans and European-Americans

**Fig. 1:**
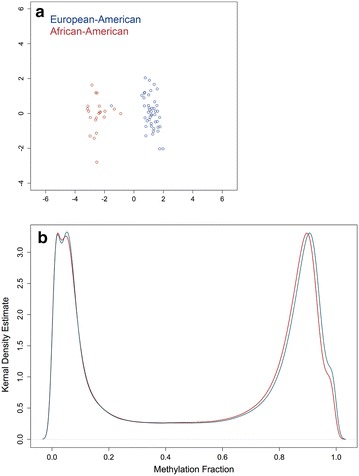
Multidimensional scaling plots of the Euclidean distances calculated using the 1000 most variable CpG sites after batch effect adjustment with ComBat and *color coded* by ethnicity (**a**). **b** Shows a kernel density estimate distribution of all *β* values before (*red line*) and after (*blue line*) batch effect adjustment. Density is a unit-less value of the probability distribution of probes within a certain range of *β* values

The Illumina Infinium HumanMethylation450 microarray interrogates the DNA methylation status of a total 485,512 probes. Differential methylation between African-American and European-American individuals was performed on 425,161 probes that remained after initial filtering. There was a clear distinction in the naïve CD4+ T cell DNA methylation profile between African-Americans and European-Americans as demonstrated in a multidimensional scaling plot of the 1000 most variable array probes after batch effect adjustment (Fig. 1). DNA methylation profile clustering can distinguish between the two ethnicities, with the exception of one European-American individual with a methylation profile that clustered with our African-American samples.

Applying a threshold of |Δβ| > 0.1 and FDR-adjusted *P* value ≤0.05, we found 306 hypomethylated CpG sites associated with 144 genes and 375 hypermethylated CpG sites associated with 164 genes in naïve CD4+ T cells isolated from African-Americans compared to European -Americans (Additional file 1: Table S1). The most hypomethylated gene was *NRGN* (Δβ = −0.41; *P* = 1.82E−05) (Table 1). This gene encodes the protein neurogranin (Ng), which is a regulator of the calcium-binding calmodulin protein [8]. Other hypomethylated genes in African-Americans included *GSTM1*, *IL32,* and *CD226*.

**Table 1:** Top 30 most hypomethylated CpG sites in naïve CD4+ T cells from healthy African-Americans compared to European Americans

CpG site	Associated gene	African-American average *β*	European-American average *β*	Δβ	*P* value	Location (chromosome: HG19 position)
cg26069044	*NRGN*	0.32	0.73	−0.41	1.82E−05	11:124613956
cg19651115		0.33	0.71	−0.37	1.20E−10	12:11700343
cg18232235		0.54	0.88	−0.34	3.61E−07	12:11700321
cg15131258	*C20orf71*	0.38	0.69	−0.31	5.45E−10	20:31805182
cg11738485	*HOOK2*	0.15	0.45	−0.31	8.55E−03	19:12877000
cg22068400		0.22	0.52	−0.31	9.56E−07	2:90016264
cg04193820	*RNF135*	0.17	0.46	−0.30	5.48E−12	17:29297414
cg08896939	*RNF135*	0.20	0.49	−0.29	2.15E−10	17:29297380
cg27436995	*FBXL16*	0.36	0.64	−0.29	1.54E−03	16:743998
cg17920646		0.39	0.67	−0.29	8.67E−04	8:216578
cg18136963		0.13	0.41	−0.28	1.54E−03	6:139013146
cg10578777		0.57	0.84	−0.27	2.07E−04	12:7781093
cg27415324		0.59	0.85	−0.27	1.38E−07	22:17901078
cg06864789		0.19	0.45	−0.26	9.11E−04	6:139012992
cg24506221	*GSTM1*	0.07	0.32	−0.26	2.94E−03	1:110230401
cg06417478	*HOOK2*	0.16	0.41	−0.25	3.19E−03	19:12876846
cg04554929		0.10	0.35	−0.25	3.27E−05	8:105342491
cg06202470		0.68	0.93	−0.24	2.13E−07	12:11700489
cg25828445		0.57	0.81	−0.24	3.08E−04	12:7781288
cg00817464	*XPNPEP1*	0.42	0.65	−0.24	4.65E−03	10:111662876
cg05886698	*DDX53*	0.67	0.90	−0.24	1.44E−06	X:23018110
cg24536782		0.36	0.60	−0.24	3.65E−03	8:216659
cg00239353	*IL32*	0.51	0.74	−0.23	1.52E−08	16:3115133
cg04752818	*INTS1*	0.63	0.87	−0.23	3.35E−06	7:1536622
cg08477332	*S100A14*	0.22	0.45	−0.23	1.47E−03	1:153590243
cg02025737		0.61	0.84	−0.23	2.46E−03	15:33384751
cg10044179	*C21orf81*	0.28	0.50	−0.22	2.43E−05	21:15352983
cg15641364	*TAGLN2*	0.17	0.40	−0.22	2.71E−07	1:159892206
cg01280390	*CSGALNACT1*	0.27	0.49	−0.22	4.86E−05	8:19363452
cg16154810	*CERK*	0.24	0.46	−0.22	6.06E−03	22:47135258

The most hypermethylated CpG site in African-Americans (Δβ = 0.39; *P* = 1.99E−08) was not associated with a gene. Other hypermethylated loci were associated with *CCS* (Δβ = 0.36; *P* = 4.78E−05) and *DUSP22* (Δβ = 0.25; *P* = 3.16E−04). *CCS* encodes a copper chaperone for super oxide dismutase that delivers copper ions to copper-dependent enzymes [9]. *DUSP22* encodes a protein tyrosine phosphatase [10].

### Gene ontologies associated with apoptosis are overrepresented in hypomethylated CpG sites in African-Americans

Using the functional annotation database DAVID, we explored biological processes and cellular component gene ontologies in genes associated with hypomethylated CpG sites in healthy African-Americans. We found genes related to regulating and promoting cellular apoptosis to be enriched among hypomethylated genes with the most significant being “positive regulation of apoptosis” (GO:0043065, FDR = 8.4 %) (Table 2). Hypermethylated CpG sites in African-American naïve CD4+ T cells were found to be enriched for gene ontologies related to the regulation of muscle adaptation and contraction (GO:0043502, FDR = 4.9 %; GO:0006936, FDR = 5.2 %), and did not appear to be related to naïve CD4+ T cell activity or growth.

**Table 2:** Biological process and cellular component gene ontologies represented in hypomethylated CpG sites in naïve CD4+ T cells from healthy African-Americans

GO term	GO term ID	Fold enrichment	False discovery rate (%)	Gene count	Genes
Positive regulation of apoptosis	GO:0043065	3.0	8.4	10	*TNFRSF10A, COL18A1, CDKN1A, AHRR, UNC13D, RIPK1, GRIN1, AKAP13, VAV2, PRODH*
Positive regulation of programmed cell death	GO:0043068	3.0	8.7	10	*TNFRSF10A, COL18A1, CDKN1A, AHRR, UNC13D, RIPK1, GRIN1, AKAP13, VAV2, PRODH*
Regulation of kinase activity	GO:0043549	3.3	9.0	9	*TNFRSF10A, NCK2, CDKN1A, DAB1, ADCY9, RIPK1, CD81, CERK, VAV2*
Positive regulation of cell death	GO:0010942	3.0	9.0	10	*TNFRSF10A, COL18A1, CDKN1A, AHRR, UNC13D, RIPK1, GRIN1, AKAP13, VAV2, PRODH*
Regulation of transferase activity	GO:0051338	3.2	11.3	9	*TNFRSF10A, NCK2, CDKN1A, DAB1, ADCY9, RIPK1, CD81, CERK, VAV2*
Positive regulation of kinase activity	GO:0033674	4.0	12.6	7	*TNFRSF10A, DAB1, ADCY9, RIPK1, CD81, CERK, VAV2*
Regulation of leukocyte activation	GO:0002694	4.7	13.1	6	*NCK2, CDKN1A, UNC13D, AP3D1, CD226, RC3H1*
Positive regulation of transferase activity	GO:0051347	3.8	14.9	7	*TNFRSF10A, DAB1, ADCY9, RIPK1, CD81, CERK, VAV2*
Regulation of cell activation	GO:0050865	4.5	16.0	6	*NCK2, CDKN1A, UNC13D, AP3D1, CD226, RC3H1*
Induction of apoptosis	GO:0006917	3.3	16.4	8	*TNFRSF10A, CDKN1A, AHRR, UNC13D, RIPK1, AKAP13, VAV2, PRODH*
Induction of programmed cell death	GO:0012502	3.3	16.6	8	*TNFRSF10A, CDKN1A, AHRR, UNC13D, RIPK1, AKAP13, VAV2, PRODH*
Regulation of cell communication	GO:0010646	2.0	17.2	16	*PLAT, GNAI3, KLK5, GRIN1, AKAP13, RGNEF, VAV2, ARHGEF10, TNFRSF10A, NCK2, ADAP2, RIPK1, ZDHHC13, CD81, CD226, KNDC1*

### Hypomethylated genes in African-Americans are enriched in genes related to autoimmunity and are hypomethylated in lupus patients

**Fig. 2:**
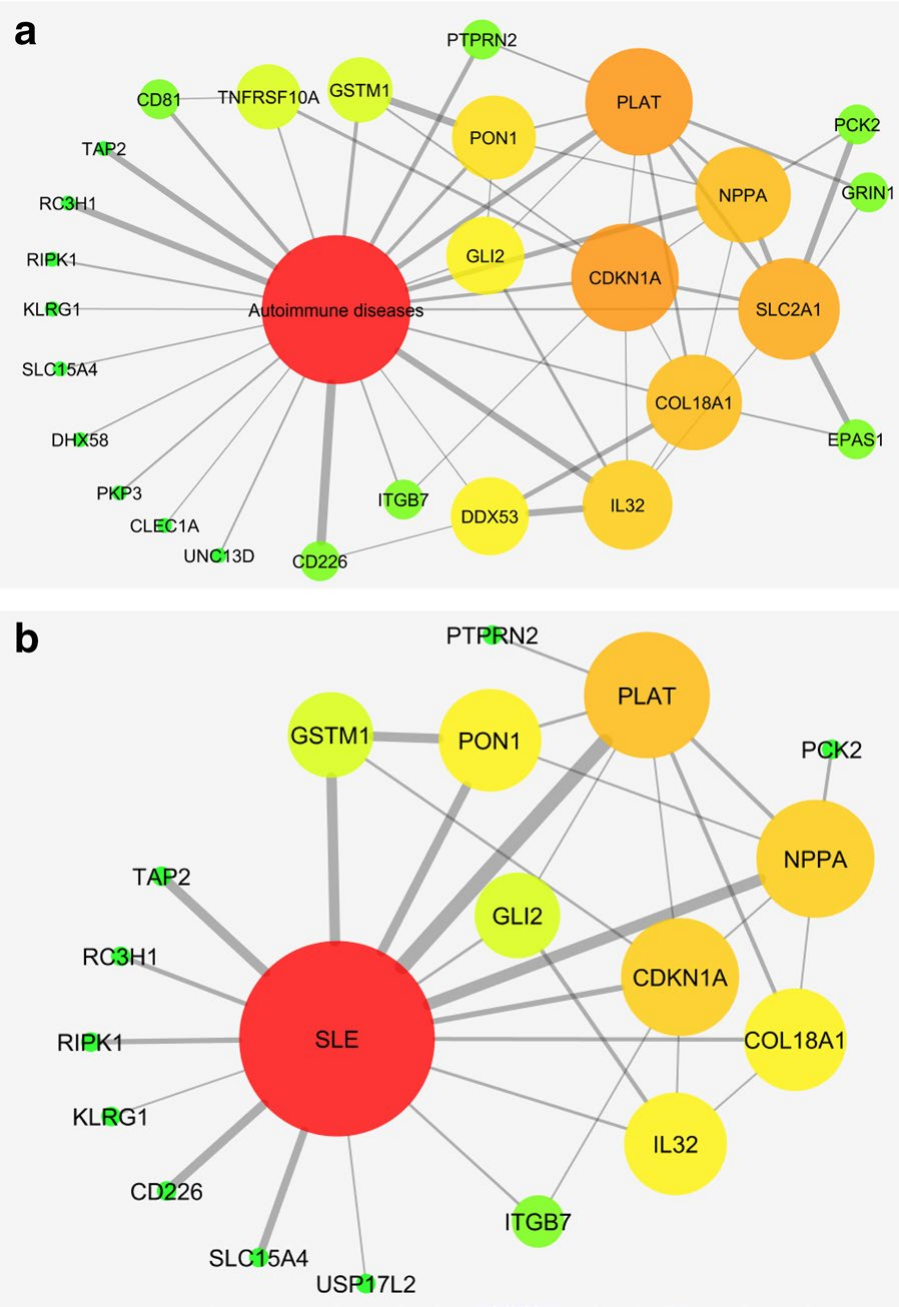
Hypomethylated genes in African-Americans that are connected in the literature to autoimmune diseases (**a**) and SLE (**b**). *Thickness of the lines* is proportional to how many publications mention each of the connections depicted, and *color* and *size* of the nodes are proportional to how many connections each node has in the network (*red* = most, *green* = fewest)

As pro-apoptotic gene ontologies were associated with hypomethylated genes in African-Americans, and because autoimmune diseases such as lupus tend to be more common and more severe in African-Americans, we investigated if hypomethylated genes in naïve CD4+ T cells in African-Americans are enriched for autoimmunity-related genes. Using the literature mining software IRIDESCENT [11–13], which scans all published MEDLINE abstracts for co-occurring terms (and their synonyms), we identified how many of these 144 genes had been published with the terms “autoimmune diseases” and “SLE”. We found 27/144 hypomethylated genes had been associated with “autoimmune diseases” (*P* < 0.005), and 18/144 associated with “SLE” (*P* < 0.037) within MEDLINE abstracts (Fig. 2). Each gene needed to be co-mentioned a minimum of three times with each query term to be considered “associated”. To validate that the CpG sites within the 18 lupus-related genes that are hypomethylated in healthy African-Americans compared to European-Americans are also differentially methylated between the two ethnicities in lupus, we compared DNA methylation levels across these sites between African-American and European-American lupus patients. We demonstrate that the majority of these CpG sites remain differentially methylated between the two ethnicities during disease, including multiple CpG sites in the cytokine gene *IL32* promoter region (Table 3).

**Table 3:** Differential DNA methylation between African-American and European-American lupus patients in CpG sites identified to be differentially hypomethylated in healthy African-Americans and located in genes connected to lupus using IRIDESCENT literature mining analysis

CpG site	Associated gene	African-American average *β*	European-American average *β*	Δβ	*P* value	Location (chromosome: HG19 position)
cg12080831	*GLI2*	0.35	0.47	−0.13	7.92E−14	2:121697428
cg23112188	*PCK2*	0.31	0.46	−0.15	3.05E−12	14:24563095
cg22816218	*RIPK1*	0.71	0.85	−0.13	3.05E−12	6:3091462
cg12091331	*PLAT*	0.64	0.74	−0.10	3.05E−12	8:42065314
cg15844419	*COL18A1*	0.69	0.81	−0.12	1.35E−08	21:46850665
cg27509823	*RC3H1*	0.74	0.82	−0.08	1.18E−07	1:173930183
cg06193043	*NPPA*	0.64	0.74	−0.10	5.32E−06	1:11908199
cg00471190	*IL32*	0.23	0.36	−0.13	6.86E−06	16:3115809
cg00239353	*IL32*	0.53	0.72	−0.19	7.62E−06	16:3115133
cg20135711	*ITGB7*	0.70	0.80	−0.10	2.13E−05	12:53592259
cg17330251	*PON1*	0.49	0.62	−0.13	9.24E−05	7:94953956
cg23813257	*IL32*	0.24	0.32	−0.08	1.06E−04	16:3115286
cg01874867	*PON1*	0.45	0.57	−0.12	1.35E−04	7:94954059
cg19678392	*PON1*	0.55	0.66	−0.11	1.70E−04	7:94953810
cg20768743	*CD226*	0.17	0.24	−0.07	2.24E−04	18:67624846
cg04871131	*PON1*	0.62	0.72	−0.10	3.28E−04	7:94954202
cg08978665	*IL32*	0.31	0.40	−0.10	4.00E−04	16:3115707
cg09035930	*SLC15A4*	0.67	0.77	−0.11	7.40E−04	12:129282057
cg20119798	*PON1*	0.50	0.61	−0.10	7.68E−04	7:94954144
cg22216157	*PTPRN2*	0.68	0.79	−0.10	7.68E−04	7:157643037
cg24425727	*CDKN1A*	0.28	0.35	−0.06	2.81E−02	6:36645648
cg04548204	*KLRG1*	0.37	0.45	−0.08	5.99E−02	12:9162872
cg27473997	*USP17*	0.73	0.78	−0.05	9.89E−02	4:9355351
cg22798247	*TAP2*	0.84	0.86	−0.02	9.89E−02	6:32807372
cg18938907	*GSTM1*	0.28	0.22	0.05	3.83E−01	1:110230456
cg11680055	*GSTM1*	0.23	0.25	−0.02	5.38E−01	1:110230252
cg24506221	*GSTM1*	0.19	0.21	−0.02	7.71E−01	1:110230401
cg10950028	*GSTM1*	0.39	0.40	0.00	9.09E−01	1:110230633

### Gene co-regulation analysis

**Fig. 3:**
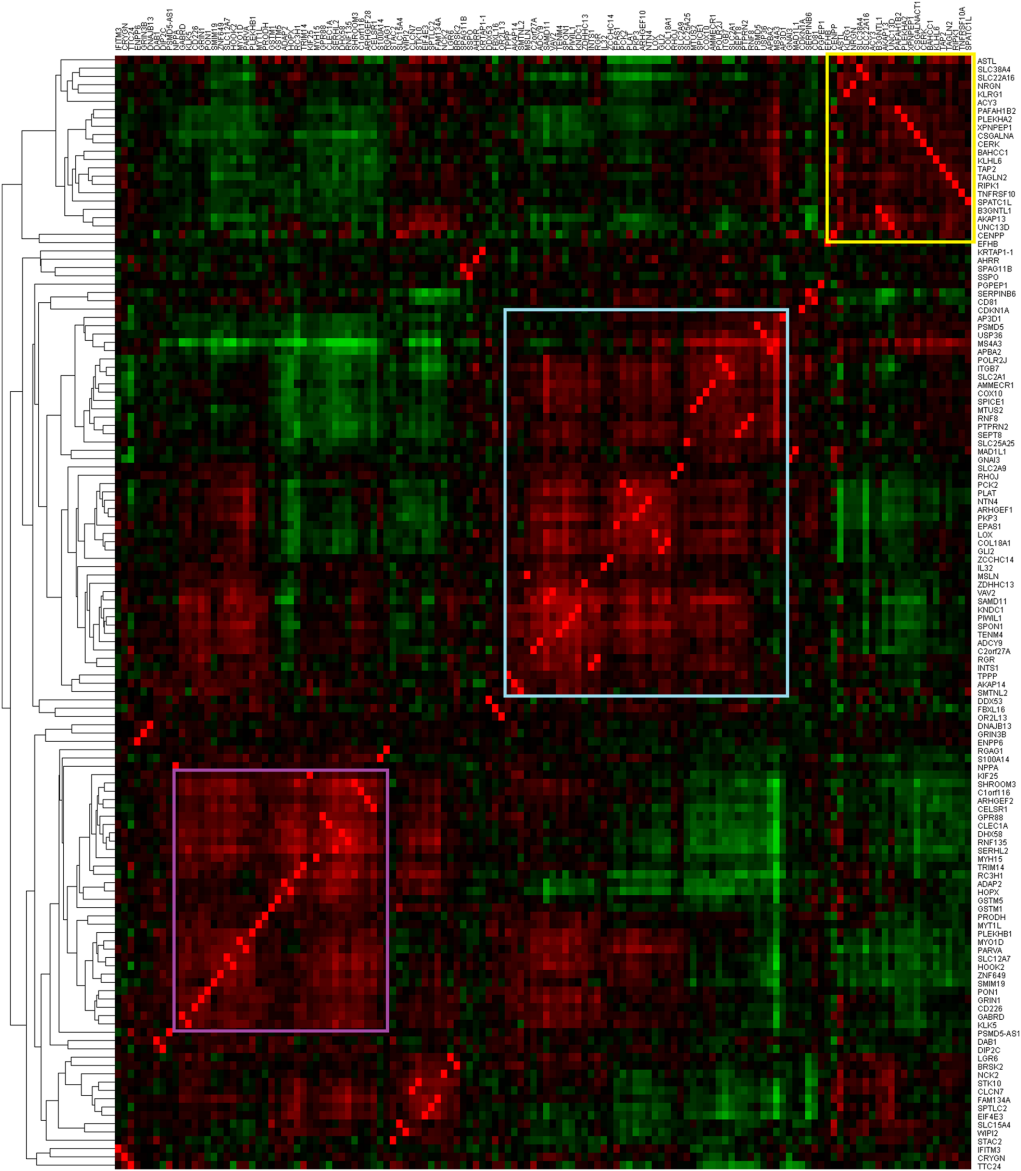
Analysis of the transcriptional correlation of 144 genes hypomethylated in African-American naïve CD4+ T cells was conducted by calculating Pearson’s correlation coefficients of the reported transcriptional fold changes across 3900 human 2-color microarray data sets. Correlation values range from 1.0 (perfect positive correlation represented in *red*) to −1.0 (perfect negative correlation represented in *green*). Three blocks of transcriptionally co-regulated genes can be seen in the *colored squares*. Using literature mining, the highest scoring themes among the genes in the *yellow*, *blue* and *purple* blocks are the cytokine IL-2, cell migration, and the glutathione S-transferase gene *GSTO1*, respectively

To further characterize differential epigenetic accessibility in naïve CD4+ T cells between African-Americans and European-Americans, we investigated the transcriptional co-regulation of the 144 hypomethylated genes in African-Americans using a meta-analysis of 3900 human gene expression microarrays [14]. Pair-wise gene–gene correlations of the 144 genes revealed three tightly co-regulated gene blocks (Fig. 3). Literature mining analysis for commonality between the genes within each block, using IRIDESCENT, revealed general relationships to IL-2 signaling, cell migration, and the glutathione S-transferase gene *GSTO1*, for the gene groups in blocks 1, 2 and 3, respectively (Fig. 3).

### *Cis*-acting genetic variants accounts for some population-specific methylation differences

**Fig. 4:**
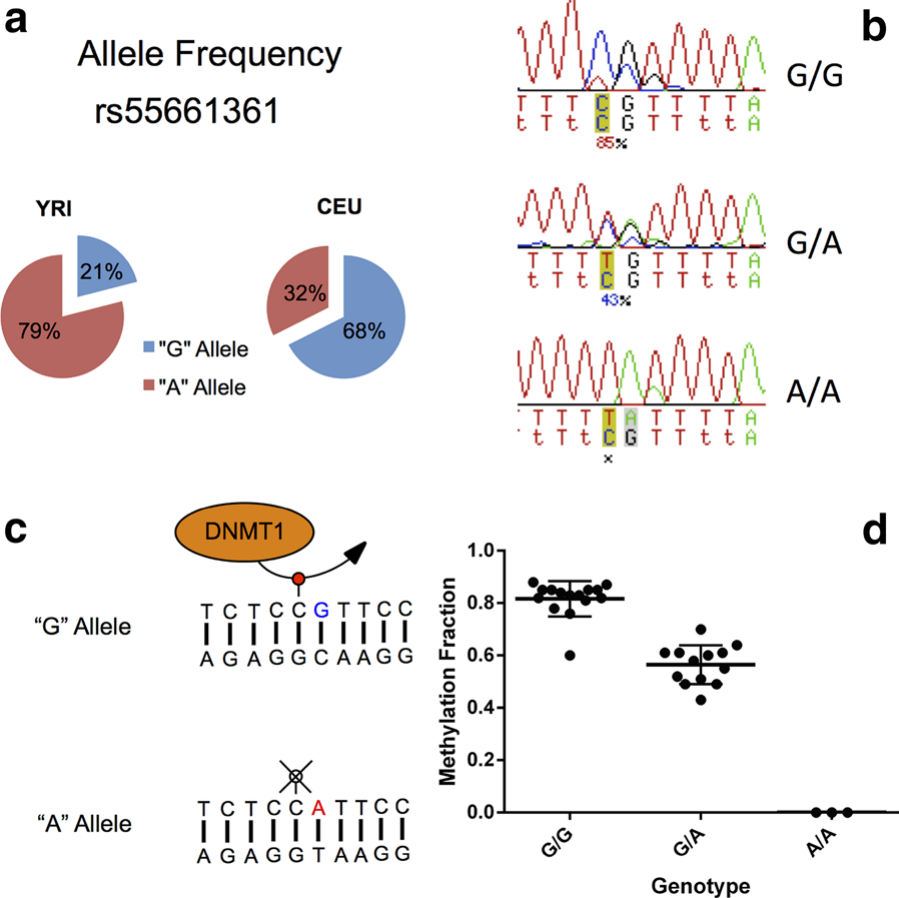
The CpG-SNP rs55661361 (A > G) in *NRGN* has an allele frequency that differs between CEU and YRI populations (**a**). Bisulfite sequencing of the CpG-SNP region in healthy individuals (**b**) revealed three distinct methylation categories based on being heterozygous or homozygous for the disruptive A allele [high methylation (G/G); intermediate methylation (G/A); low methylation (A/A)]. A cartoon (**c**) demonstrating the effect of the A allele on cytosine methylation by DNMT1. Average DNA methylation levels in *NRGN* stratified by genotype in 31 healthy individuals included in the study (**d**). Genotype, average methylation ± SD, *N*: *G/G*, 0.82 ± 0.07, *N* = 15; *G/A*, 0.56 ± 0.07, *N* = 13; *A/A*, 0 ± 0, *N* = 3

To investigate if *cis*-acting genetic variants might explain at least some of the DNA methylation differences between African-American and European-American individuals, the presence of single-nucleotide polymorphisms (SNPs) with a minor allele frequency of >1 % located within the top 30 hypomethylated CpG dinucleotides in African-Americans was determined using the UCSC Genome Browser. These SNPs located beyond the array probe sequences do not influence probe hybridization and the technical ability of probes to accurately measure DNA methylation. The top 30 hypomethylated CpG sites included four SNPs (substitutions or insertion–deletions), three of which were associated with genes including *NRGN, BPIFA3* (*C20orf71*) and *INTS1*. Rs55661361 is an intragenic SNP located in the *NRGN* CpG site most hypomethylated in African-Americans. The ancestral A allele changes the CpG site in *NRGR* to a CpA site, which would prevent the methylation of the cytosine residue in this locus. The ancestral A allele has a frequency of 79 and 37 % in the Yoruba from Ibadan, Nigeria (YRI) and the European-Americans from Utah, USA (CEU) populations, respectively. This suggests that loss of DNA methylation in this CpG site in African-Americans might be explained by this genetic variation. To confirm this, we performed bisulfite DNA sequencing in 10 African-American and 21 European-American healthy individuals to determine the genotype–methylation relationship in this locus. We found a distinct pattern of association between the genotype of rs55661361 and the average DNA methylation of *NRGN* (Fig. 4). Individuals in this study can be categorized into high methylation, intermediate methylation, and low methylation groups representing three genotypes: G/G, G/A and A/A in rs55661361, respectively. The high methylation group (G/G) included 15 individuals (13 EA; 2 AA; average  % methylation = 81.6), the intermediate methylation group (G/A) included 13 individuals (8 EA; 5 AA; average  % methylation = 56.5), and the low methylation group (A/A) included 3 individuals (0 EA; 3 AA; average  % methylation = 0.0) (Fig. 4). Therefore, the DNA methylation difference between the two ethnicities on this CpG site can be completely explained by the genotype on rs55661361.

### Patch methylation of the IL32 promoter region suppresses gene expression in vitro

**Fig. 5:**
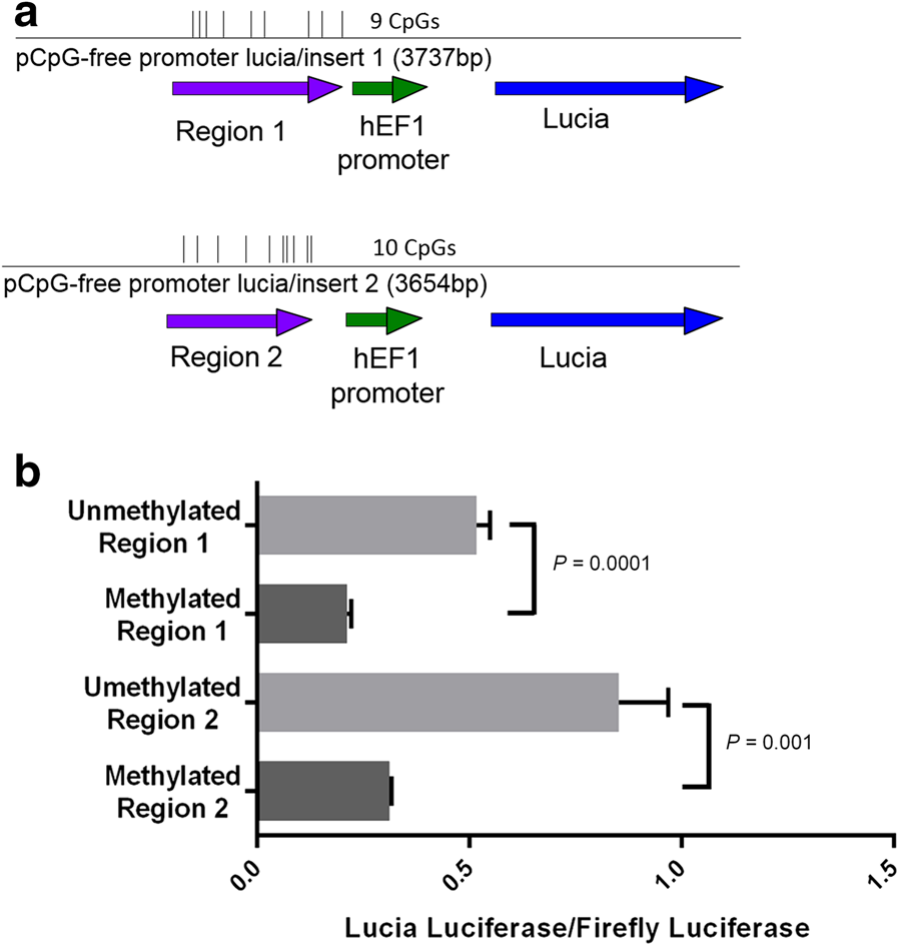
Using a luciferase reporter vector with an inserted promoter/5'-untranslated regions from *IL32* shows a significant difference in luciferase expression between fully methylated and unmethylated regions as measured by a ratio of Lucia luciferase over firefly luciferase luminescence. A cartoon (**a**) of the linearized plasmid showing the location of the *IL32* promoter/5'-untranslated region inserts in the Lucia Luciferase reporter vector and the number of CpG sites present in each insert. The expression of the Lucia luciferase reported as a ratio of Lucia/firefly luciferase (**b**)

To confirm that DNA methylation changes we report are transcriptionally relevant, we tested if the differentially methylated regions we observed in *IL32* can influence gene expression using in vitro reporter assays. Both of the upstream promoter/5'-untranslated regions hypomethylated in African-Americans compared to European-Americans, in healthy individuals and in lupus patients, displayed decreased luminescence after being fully methylated in vitro and inserted upstream of a Lucia luciferase reporter gene compared to an identical sequence that remained unmethylated. The Lucia/firefly luciferase ratio for the unmethylated Region 1 insert (−230 to +232 from *IL32* transcription start site) was 2.43-fold greater than the methylated insert (*P* = 0.0001). The ratio for unmethylated Region 2 insert (+194 to +573 from *IL32* transcription start site) was 2.74-fold greater than the methylated insert (*P* = 0.001) (Fig. 5).

## Discussion

We present the first comparison of the genome-wide DNA methylation status of peripheral naïve CD4+ T cells in healthy African-American and European-American individuals. Site-specific methylation differences between ethnicities was due in part to the presence of genetic variants that alter the methylation status of CpG dinucleotides. One mechanism behind these differences is the disruption of the CpG dinucleotide motif recognized by DNA methyltransferase 1 enzyme (DNMT1) [15]. DNMT1 recognizes hemi-methylated CpG dinucleotides during DNA replication and methylates the unmethylated CpG dinucleotide on the newly synthesized DNA strand. The effects of these polymorphisms in the genome would be a reduction in average DNA methylation of CpG sites in individuals with the disruptive allele. Differences in average DNA methylation would be greatest for CpG-SNPs that have significantly different allele frequencies between populations. The occurrence of CpG-SNPs and their effect on allele-specific methylation regions is highly population and cell-type specific. One study reported that 38–88 % of allele-specific methylation regions measured across 16 human adult and pluripotent cell lines were present solely due to CpG-SNPs. This allele-specific methylation was also found to extend beyond the individual CpG site in some instances, resulting in reduced methylation at nearby sites that contained no overlapping SNPs [16].

The most hypomethylated CpG site in African-Americans in this study in *NRGN* is a prime example of the effect genetic variation can have on DNA methylation. The presence of the A allele is associated with reduced methylation and is the major allele in African-Americans, but the minor allele in European-Americans. We demonstrated a stratification of African-American and European-American individuals by methylation status and rs55661361 genotype, with African-Americans having significant reduction in average DNA methylation in this locus compared to European-Americans. In addition to the *NRGN* locus, 3 others of the top 30 most hypomethylated CpG sites included disruptive SNPs. The population-specific frequency of these disruptive alleles varies between CEU and YRI populations. There are likely more CpG-SNPs across the genome that alter DNA methylation and account for differences between populations.

Neurogranin (Ng), encoded by *NRGN*, regulates calcium-dependent apoptosis in mature T cells [17]. These IL-2-dependent cells express Ng and interact with the calcium-binding protein calmodulin. Devireddy et al. proposed an interaction model in which an IL-2 deprived environment increases Ng expression and Ng-calmodulin interaction displaces calmodulin-bound Ca^2+^ and thus raises the intercellular Ca^2+^ concentration, promoting apoptosis. It is worth noting that lupus T cells are characterized by defective IL-2 expression, enhanced calcium influx, and increased apoptosis, and that lupus is more common in African-Americans compared to European-derived populations [18]. Further, recent reports suggest increased Ng expression in PBMCs of lupus patients [19, 20].

Apoptosis was found to be a recurring theme among genes associated with hypomethylated CpG sites in naïve CD4+ T cells from African-American individuals.

Gene ontology terms for the hypomethylated genes show an enrichment of cellular apoptosis driven by the hypomethylation of genes such as *TNFRSF10A, COL18A1, CDKN1A, AHRR, UNC13D, RIPK1, GRIN1, AKAP13, VAV2,* and *PRODH*. Tumor necrosis factor receptor superfamily, member 10a (or TRAILR1) is a cell surface protein encoded by *TNFRSF10A*. It recognizes extracellular Apo-2L (or TRAIL) and acts as a signal for the downstream recruitment of pro-apoptotic caspases in the cell [21].

Four CpG sites upstream of the cytokine gene *IL32* were hypomethylated in African-American individuals independently of any known SNP. An 803-bp region (chr16:3115083–3115886; HG19) immediately upstream of and including the *IL32* gene transcription start site and its 5'-untranslated region contained these four CpG sites. These sites were cloned with their surrounding sequence and inserted into a CpG-free luciferase reporter vector. The unmethylated and in vitro methylated regions were compared and showed a significant reduction in the expression of luciferase in transfected HEK 293 cells when all CpG sites were methylated. These regions contain regulatory elements that are influenced by DNA methylation and could regulate the expression of *IL32* in peripheral T cells. IL-32 is a cytokine that induces the production of MIP-2, IL-8 and TNF in monocytic cell lines [22]. IL-32 (primarily IL-32β) expression in activated CD4 + T cells was also associated with increased apoptosis and was presumed to play a role in activation-induced cell death [23]. The hypomethylation of apoptosis-related genes could indicate an epigenetic susceptibility to apoptosis and might contribute to the pathogenesis of diseases in which T cell apoptosis is an associated factor. A significant increase in T cell apoptosis has been reported in SLE patients compared to controls [24]. Apoptosis was also increased in active (SLE Disease Activity Index >4) SLE patients compared to inactive patients. Taken together, hypomethylation of pro-apoptotic genes in naïve CD4+ T cells from healthy African-Americans may contribute to the increased frequency and severity of lupus in African-Americans.

The glutathione S-transferase mu 1 protein (GSTM1) encoded by *GSTM1* is involved in the metabolism of xenobiotic compounds including carcinogens and products of oxidative stress. It plays a role in processing carcinogens and has been previously associated with cancer risk [25]. There were 4 CpG sites in *GSTM1*, two within 200 bp upstream of the transcription start site, one in the 1st exon, and one in the gene body, with significant hypomethylation in African-American naïve CD4+ T cells.

We also found significant hypomethylation in *CD226* in African-American naïve CD4+ T cells. CD226 is expressed on the surface of T cells and natural killer T cells. It is involved in the activation, adhesion, cytotoxicity, and differentiation of T cells, and has previously been identified as a genetic risk locus for SLE [26]. The risk locus is located downstream of the *CD226* gene body while the demethylated CpG site is within 1500 bp of the transcription start site.

In summary, we identified significant epigenetic difference in naïve CD4+ T cells between African-American and European-American healthy individuals. These ethnicity-specific epigenetic differences are at least in part derived from *cis*-acting genetic polymorphisms. We found significant hypomethylation in pro-apoptotic genes in African-Americans, and hypomethylation of pro-inflammatory genes such as *IL32*. This epigenetic variability might play a role in increased frequency and severity of some autoimmune diseases such as lupus, which is characterized by increased T cell apoptosis. Indeed, of the 144 hypomethylated genes in African-American naïve CD4+ T cells, 18 and 27 have been previously related to lupus and autoimmune diseases, respectively. We demonstrated that the majority of these 18 lupus-related genes were also hypomethylated in African-American compared to European-American lupus patients, providing further validation and disease-relevance of the ethnicity-specific DNA methylation changes reported in this study. Further work is required to determine if these hypomethylated sites exist in a transient state or are maintained as the cell develops, and whether or not they are relevant to other cell types. Exploration of the DNA methylome could help further resolve what inherent epigenetic differences in immune activity exist among populations that might influence the pathogenesis of complex heterogeneous diseases.

## Conclusion

We provide evidence for wide-spread ethnicity-specific differences in DNA methylation patterns in naïve CD4+ T cells. These differences promote an overall T cell chromatin architecture that is more permissive to autoimmunity in African-Americans compared to European-Americans. Indeed, hypomethylated loci in African-Americans are enriched in pro-apoptotic genes involved in autoimmunity. These same genes are also hypomethylated in African-American compared to European-American lupus patients. Our data also provide strong evidence that these methylation differences between ethnicities are at least in part a result of genetic variants that directly disrupt CpG dinucleotides. Therefore, inherited epigenetic susceptibility loci contribute to explaining differences in susceptibility to autoimmune diseases, including lupus, between populations.

## Methods

### Study participants

A total of 66 healthy female individuals (21 African-Americans and 45 European-Americans) were recruited for our studies. There was no significant difference in age between the African-American (mean age = 39.3 years) and European-American (mean age = 42.5 years) groups (*P* value = 0.34). All study participants signed an informed consent prior to enrollment into the study. The institutional review boards at the University of Michigan and the Henry Ford Health System approved this study. To validate the relevance of detected ethnicity-specific DNA methylation differences in an autoimmune disease, a group of age-matched 21 African-American and 42 European-American female lupus patients were studied. All patients met the American College of Rheumatology classification criteria for SLE [27].

### Isolation of naïve CD4+ T cells

Whole blood was collected at the time of enrollment and processed within 6 h of collection. Peripheral blood mononuclear cells were isolated using Ficoll density gradient centrifugation media (GE Healthcare Biosciences, USA) followed by indirect isolation of untouched naïve CD4+ T cells using the Naïve CD4+ T cell Isolation Kit II (Miltenyi Biotec Inc., USA). DNA was isolated using the DNeasy Blood and Tissue Kit (Qiagen, USA) and DNA concentration was determined by spectrophotometry on the NanoDrop 2000 (Thermo Scientific, USA). Flow cytometry was used to confirm purity of isolated naïve CD4+ T cells using CD4 and CD45RA fluorochrome-conjugated antibodies (BioLegend, USA).

### DNA methylation analysis

500 ng of DNA from each sample was bisulfite converted using the EZ DNA Methylation™ Kit (Zymo Research, USA) as previously described [6]. DNA methylation was evaluated by hybridizing bisulfite-converted DNA to the Infinium HumanMethylation450 BeadChips (Illumina, USA) according to manufacturer’s directions. The Infinium array interrogates over 485,000 methylation sites across the human genome, covering 96 % of CpG islands, shores and flanking regions as well as 99 % of RefSeq genes with an average of 17 CpG sites per gene region. The array covers the promoter, 5′-untranslated region, first exon, gene body, and 3′-untranslated region of each gene. Array hybridization of the bisulfite-converted DNA was performed at the University of Michigan DNA Sequencing Core.

### Methylation data processing and analysis

DNA methylation data analysis was performed using the minfi (1.12.0) statistical analysis package in the R environment (3.1.2) [28, 29] (Additional file 1: Figure S1). Minfi is an analysis suite for Infinium HumanMethylation450 data that allows user flexibility in preprocessing, quality control, and identification of differentially methylated regions. Raw data were imported into minfi as a single object of red and green channel intensity values and sample phenotype information. Preprocessing of these channel values was accomplished using a method included in minfi that is equivalent to the data preprocessing method in the Illumina GenomeStudio Methylation Module that converts the red and green channel intensities into methylated and unmethylated values. This included correction for background signal and normalization of each channel to control probes included on each array. An array was chosen at random for normalization. After preprocessing, 306 probes with a detection *P* value >0.05 in >20 % of the probes were removed from analysis. 60,045 probes were identified as having a SNP within 10 base pairs of the interrogated nucleotide and were removed from analysis as well. Using minfi’s “fixMethOutliers” command, extreme values in the methylated and unmethylated channels were identified separately. An intensity cutoff value was calculated by first log_2_(*x* + 0.5) transforming all intensity values and then calculating the median intensity and median intensity absolute deviation. An intensity cutoff was defined as the median + (−3 * median absolute deviation). Any intensity less than the cutoff value was considered an outlier, and adjusted to be equal to the intensity cutoff. The methylated and unmethylated probe values were used to calculate both a Beta (*β*) and *M* value ratio. *β* values are calculated using the formula: $\beta = \frac{\text{Methylated}}{{{\text{Methylated}}\; + \;{\text{Unmethylated}}\; +\; 100}}$ and *M* values are calculated as the logit(*β*).

Batch effect correction was performed using the ComBat script available as part of the sva (3.12.0) package [30]. ComBat utilizes a parametric empirical Bayes method to first standardize means and variances across batches for normalized data. Next it uses the distribution of the standardized data to determine batch effect parameters, and finally, uses the empirically determined batch effect estimators to adjust the data. Individual Infinium microarray chips used were input into ComBat as batches. *M* values were calculated from normalized *β* values and used as input for ComBat, as it assumes a normal distribution of the data, and the batch effect-adjusted matrix of data was converted back into *β* values for further analysis.

After adjusting for batch effect, the delta *β* (Δβ) of each probe between the African-American and European-American groups was calculated as $\varDelta \beta = {\text{Mean}} \beta \left( {{\text{African}} - {\text{American}}} \right) - {\text{Mean}} \beta ({\text{European}} - {\text{American}})$. A two-sided Student’s *t* test was performed assuming equal variances of the mean $\beta$ in each group to calculate a *P* value adjusted for false discovery rate (FDR). Further analysis was limited to probes with an FDR-adjusted *P* value of less than or equal to 0.05 and a |Δβ| greater than 0.1.

### Gene ontology and bioinformatics analysis

Gene ontologies (GO) identification of hypo- and hypermethylated probes was performed using the Database for Annotation, Visualization and Integrated Discovery (DAVID v6.7) [31, 32]. Search parameters used in DAVID were a minimum gene group membership of 2, a Modified Fisher Exact *P* value (EASE Score) maximum of 0.1, and the human genome as background. Further bioinformatics analysis was conducted using IRIDESCENT [12].

### Bisulfite DNA sequencing

Confirmation of the methylation status reported by the BeadChip array for each donor was conducted by first bisulfite converting 100 ng of genomic DNA from 31 samples (21 EA; 10 AA) as described earlier in this paper. Polymerase chain reaction (PCR) was used to amplify a 581-bp region of *NRGN* (chr11:124613509–124614089; HG19) using forward (5′-GAATGTGAAGTTTAGGAAATGAGGT-3′) and reverse (5′-TTCAACCTAATAATAATCAAACCCACCTCT-3′) primers added to Zymo*Taq*™ PreMix (Zymo Research, USA). PCR thermocycler settings were as follows: DNA denaturation at 95 °C for 10 min followed by 40 cycles at 95 °C for 30 s, then 56 °C for 40 s, and finally 72 °C for 1 min. PCR amplification of the desired region was confirmed by electrophoresis of an aliquot from each product on a 2.0 % agarose gel at 93 V for 1 h. The remaining PCR product was prepared for sequencing using the DNA Clean and Concentrator-5 Kit (Zymo Research, USA) following manufacturer’s directions and DNA concentration was determined using the NanoDrop 2000 (Thermo Scientific, USA). Sequencing was performed on an ABI Model 3730XL sequencing platform. Average DNA methylation and genotypes were determined using Epigenetic Sequencing MEthylation (ESME) analysis software [33]. *β* values determined by ESME and the HumanMethylation450 array showed a high, positive correlation (*R*^2^ = 0.8217).

### Co-regulation analysis of hypomethylated genes

The co-expression of 144 hypomethylated genes was determined using 3900 processed, 2-color human gene expression microarrays retrieved from the Gene Expression Omnibus as previously described [14]. Global gene–gene pair co-expression was quantified using Pearson’s correlation coefficients.

### Ligation of IL32 promoter regions into luciferase reporter vector

Two regions containing CpG sites of interest within the *IL32* promoter/5'-untranslated regions were amplified from human genomic DNA. Region 1 (chr16:3115083–3115545; HG19) (forward primer: 5′-TTTGAGCTCCCTATCTTGTCCCACAGGTAGA-3′, reverse primer: 5′-AGGTACTCGAGACAGACAGAGACAGAGACAGAG-3′) contains cg00239353, cg26724967, and cg23813257 and region 2 (chr16:3115507–3115886; HG19) (forward primer: 5′-CCCGAGCTCTCTCTGTCTGTCTCTGTCTCTG-3′, reverse primer: 5′-ATGTACTCGAGGCACCCTTACGGTCTGTTT-3′) contains cg18350391, cg08978665, and cg00471190. These regions also contained other CpG sites not included on the Infinium array. The PCR thermocycler conditions used were as follows: 30 s at 98 °C, and 3 cycles of 98 °C for 10 s followed by 67 °C for 30 s followed by 72 °C for 20 s, and 27 cycles of 98 °C for 10 s followed by 58 °C for 30 s followed by 72 °C for 20 s, and 2 min at 72 °C. Each amplified region was Sanger sequenced to verify the correct sequence was amplified. The correctly amplified regions were ligated using T4 DNA ligase (New England BioLabs, USA) into a CpG-free Lucia luciferase reporter vector (pCpGfree-Lucia; InvivoGen, USA) (Fig. 5a) digested with *Sca*I. Both regions were inserted immediately upstream of a human *EF1* promoter. Each ligation reaction contained a 5:1 insert: vector molar ratio, and was incubated overnight at 16 °C, then incubated 30 min at room temperature and heat inactivated at 65 °C.

The plasmids were transformed into *E. coli* GT115 (InvivoGen, USA) using the Mix and Go *E. coli* transformation kit (Zymo Research, USA) with 1 h of outgrowth, and grown on Zeocin selective plates and media (InvivoGen, USA). Plasmid DNA from transformed liquid cultures was extracted using the QIAprep Spin Miniprep Kit (Qiagen, USA), and the direction of each insert within the plasmid was determined by Sanger sequencing using the forward primer previously listed for each region.

### Patch methylation of IL32-promoter region

0.5 µg of each complete plasmid were methylated via M.SssI methyltransferase (New England BioLabs, USA) or mock methylated (methyltransferase omitted). Each methylation reaction contained 640 mM S-adenosylmethionine and 16U M.SssI, and was incubated overnight at 37 °C and heat inactivated at 65 °C for 20 min. The CpG-free promoter vector is modified to contain no CpG sites, so the inserted promoter regions are exclusively methylated. Complete methylation was verified by digesting 100 ng of plasmid with *Sac*I and methylation-sensitive restriction enzymes (*Hpy*CH4IV for region 1, and *Aci*I for region 2) at 37 °C for 1 h (New England BioLabs, USA). For region 1, the expected digestion product size was either 3.7 kb if methylated or 1.8 kb if unmethylated. For region 2, the expected product size was 3.7 kb if methylated or 1.6 and 1.9 kb if unmethylated. Only methylation reactions that were verified as completely methylated by gel electrophoresis were used in transfection.

### Luciferase reporter assay of methylated and unmethylated IL32 promoter regions

HEK 293 cells in an opaque white 96 well plate were transfected in triplicate with 50 ng control firefly luciferase plasmid (Promega, USA) and 50 ng of pCpGfree-Lucia constructs, using Lipofectamine 3000 (Invitrogen, USA) according to manufacturer’s instruction. The pCpGfree-Lucia constructs used were either the methylated or mock methylated plasmids containing either region 1 or region 2, as well as a vector containing no insert, and a DNA-free control. After 48 h, the luciferase activity was measured after simultaneously treating an aliquot of the supernatant media with QUANTI-Luc reagent (InvivoGen, USA) to measure Lucia luciferase activity, and treating the adherent cells with Dual-Glo Luciferase reagent (Promega, USA) to measure firefly luciferase activity, and immediately reading the resulting luminescence with a Synergy H1 microplate reader (BioTek, USA) with a gain of 100 units. The reads were background corrected to the DNA-free control, then controlled for transfection efficiency by reporting the ratio of Lucia/firefly luciferase luminescence. These ratios were compared to determine the effect of methylation on luciferase activity using a Student’s *t* test with a statistical significance threshold of *α* ≤ 0.05 (Fig. 5b).

## Additional file


10.1186/s13072-015-0037-1 Supplementary material (Table S1 and Figure S1).

## Abbreviations

SLE: Systemic lupus erythematosus
CpG: Cytosine–phosphate–guanosine
DAVID: Database of Annotation, Visualization and Integrated Discovery
GO: Gene ontology
LINE-1: Long interspersed nucleotide element 1
AA: African-American
EA: European-American
YRI: Yoruba from Ibadan
CEU: CEPH (Centre d’Etude du Polymorphisme Humain) from Utah
Ng: Neurogranin
FDR: False discovery rate
