# Supplementary material for: Ethnicity-specific epigenetic variation in naïve CD4+ T cells and the susceptibility to autoimmunity
Source: Epigenetics Chromatin. 2015 Nov 24;8:49. doi: 10.1186/s13072-015-0037-1 (PMC4659164; doi:10.1186/s13072-015-0037-1)
Supplement: Supplementary file 1 — 10.1186/s13072-015-0037-1 Supplementary material (Table S1 and Figure S1). [file 13072_2015_37_MOESM1_ESM.docx]

**Supplementary Figure 1:**

**Data Importation**

Intensity Data (.idat) files and sample phenotype data were read into the R environment by minfi as a set of red and green channel intensities

**Normalization and Background Subtraction**

Channel intensity values were background subtracted and normalized

**Data Cleanup**

All probes containing a SNP within 10bp of the probe 3’ end and/or detection *P* values > 0.05 in more than 20% of the probes were removed from further analysis

**Outlier Adjustment**

Methylated and unmethylated intensity values were identified using a cutoff of median + (-3 * median absolute deviation) of the log_2_(x+0.5) transformed values. Any values exceeding this cutoff were outliers and thresholded to equal the cutoff value

**Batch Effect Adjustment**

Outlier-adjusted methylated and unmethylated intensity values were used to calculate M-values (logit transformed β-values). These M-values were used for batch effect correction by ComBat and the newly adjusted values were transformed into β-values

**Calculation of Δβ**

Differential β-values were calculated as Δβ = 𝑀𝑒𝑎𝑛 β_African−American_ *−* 𝑀𝑒𝑎𝑛 β_European−American_. Genes with a Δβ ≥1.0 or ≤-1.0 and an FDR-adjusted t-test *P* value of <0.05 were selected for further analysis.

**Gene Ontology and Pathway Analysis**

Significantly differentially methylated genes were separated into hypomethylated (Δβ≤-1.0) and hypermethylated (Δβ≥1.0) gene sets. Gene ontology and pathway analysis were conducted using DAVID and IRIDESCENT

**Supplementary Table 1**: Differentially methylated probes in healthy African-Americans compared to healthy European-Americans. Probes were considered differentially methylated if the probe Δβ was ≥|1.0| and FDR-adjusted t-test P value was <0.05.

| **Probe ID** | **African-American Average β** | **European-American Average β** | **Δβ** | **FDR-adjusted *P* value** | **Location (Chr:HG19 Position)** | **UCSC RefGene ID** | **UCSC RefGene Group** |
| --- | --- | --- | --- | --- | --- | --- | --- |
| cg26069044 | 0.32 | 0.73 | -0.41 | 1.82E-05 | 11:124613956 | *NRGN;NRGN* | Body;Body |
| cg19651115 | 0.33 | 0.71 | -0.37 | 1.20E-10 | 12:11700343 |  |  |
| cg18232235 | 0.54 | 0.88 | -0.34 | 3.61E-07 | 12:11700321 |  |  |
| cg15131258 | 0.38 | 0.69 | -0.31 | 5.45E-10 | 20:31805182 | *C20orf71;C20orf71;C20orf71;C20orf71* | 5'UTR;1stExon;5'UTR;1stExon |
| cg11738485 | 0.15 | 0.45 | -0.31 | 8.55E-03 | 19:12877000 | *HOOK2;HOOK2* | Body;Body |
| cg22068400 | 0.22 | 0.52 | -0.31 | 9.56E-07 | 2:90016264 |  |  |
| cg04193820 | 0.17 | 0.46 | -0.30 | 5.48E-12 | 17:29297414 | *RNF135;RNF135* | TSS1500;TSS1500 |
| cg08896939 | 0.20 | 0.49 | -0.29 | 2.15E-10 | 17:29297380 | *RNF135;RNF135* | TSS1500;TSS1500 |
| cg27436995 | 0.36 | 0.64 | -0.29 | 1.54E-03 | 16:743998 | *FBXL16* | 3'UTR |
| cg17920646 | 0.39 | 0.67 | -0.29 | 8.67E-04 | 8:216578 |  |  |
| cg18136963 | 0.13 | 0.41 | -0.28 | 1.54E-03 | 6:139013146 |  |  |
| cg10578777 | 0.57 | 0.84 | -0.27 | 2.07E-04 | 12:7781093 |  |  |
| cg27415324 | 0.59 | 0.85 | -0.27 | 1.38E-07 | 22:17901078 |  |  |
| cg06864789 | 0.19 | 0.45 | -0.26 | 9.11E-04 | 6:139012992 |  |  |
| cg24506221 | 0.07 | 0.32 | -0.26 | 2.94E-03 | 1:110230401 | *GSTM1;GSTM1* | TSS200;TSS200 |
| cg06417478 | 0.16 | 0.41 | -0.25 | 3.19E-03 | 19:12876846 | *HOOK2;HOOK2* | Body;Body |
| cg04554929 | 0.10 | 0.35 | -0.25 | 3.27E-05 | 8:105342491 |  |  |
| cg06202470 | 0.68 | 0.93 | -0.24 | 2.13E-07 | 12:11700489 |  |  |
| cg25828445 | 0.57 | 0.81 | -0.24 | 3.08E-04 | 12:7781288 |  |  |
| cg00817464 | 0.42 | 0.65 | -0.24 | 4.65E-03 | 10:111662876 | *XPNPEP1;XPNPEP1;XPNPEP1* | Body;Body;Body |
| cg05886698 | 0.67 | 0.90 | -0.24 | 1.44E-06 | X:23018110 | *DDX53;DDX53* | 1stExon;5'UTR |
| cg24536782 | 0.36 | 0.60 | -0.24 | 3.65E-03 | 8:216659 |  |  |
| cg00239353 | 0.51 | 0.74 | -0.23 | 1.52E-08 | 16:3115133 | *IL32;IL32;IL32;IL32;IL32;IL32;IL32;IL32* | TSS1500;TSS200;TSS1500;TSS1500;TSS200;TSS200;TSS200;TSS1500 |
| cg04752818 | 0.63 | 0.87 | -0.23 | 3.35E-06 | 7:1536622 | *INTS1* | Body |
| cg08477332 | 0.22 | 0.45 | -0.23 | 1.47E-03 | 1:153590243 | *S100A14* | TSS1500 |
| cg02025737 | 0.61 | 0.84 | -0.23 | 2.46E-03 | 15:33384751 |  |  |
| cg10044179 | 0.28 | 0.50 | -0.22 | 2.43E-05 | 21:15352983 | *C21orf81* | TSS1500 |
| cg15641364 | 0.17 | 0.40 | -0.22 | 2.71E-07 | 1:159892206 | *TAGLN2* | 5'UTR |
| cg01280390 | 0.27 | 0.49 | -0.22 | 4.86E-05 | 8:19363452 | *CSGALNACT1;CSGALNACT1;CSGALNACT1* | 5'UTR;Body;5'UTR |
| cg16154810 | 0.24 | 0.46 | -0.22 | 6.06E-03 | 22:47135258 | *CERK* | TSS1500 |
| cg17518825 | 0.54 | 0.76 | -0.22 | 1.84E-08 | 2:46523461 | *EPAS1* | TSS1500 |
| cg04657146 | 0.18 | 0.40 | -0.22 | 4.05E-03 | 19:12876947 | *HOOK2;HOOK2* | Body;Body |
| cg15465743 | 0.53 | 0.75 | -0.21 | 3.56E-04 | 11:1413145 | *BRSK2* | Body |
| cg07185983 | 0.40 | 0.61 | -0.21 | 3.02E-04 | 6:139012860 |  |  |
| cg10070864 | 0.53 | 0.75 | -0.21 | 1.45E-08 | 15:57664490 |  |  |
| cg01347228 | 0.13 | 0.33 | -0.20 | 1.58E-12 | 17:29297391 | *RNF135;RNF135* | TSS1500;TSS1500 |
| cg15501526 | 0.40 | 0.61 | -0.20 | 6.17E-05 | 10:2543763 |  |  |
| cg26536949 | 0.52 | 0.72 | -0.20 | 4.11E-02 | 17:57053 |  |  |
| cg06562718 | 0.65 | 0.85 | -0.20 | 2.08E-04 | 8:1789169 | *ARHGEF10* | 5'UTR |
| cg09549987 | 0.38 | 0.58 | -0.20 | 3.73E-02 | 8:7320532 | *SPAG11B;SPAG11B;SPAG11B;SPAG11B;SPAG11B* | Body;Body;Body;Body;Body |
| cg03329597 | 0.29 | 0.48 | -0.20 | 2.81E-02 | 3:108125523 | *MYH15* | Body |
| cg21484985 | 0.63 | 0.82 | -0.20 | 1.21E-05 | 14:70690525 |  |  |
| cg18938907 | 0.14 | 0.34 | -0.19 | 3.54E-02 | 1:110230456 | *GSTM1;GSTM1;GSTM1;GSTM1* | 1stExon;5'UTR;1stExon;5'UTR |
| cg05275182 | 0.64 | 0.83 | -0.19 | 9.51E-07 | 16:29179176 |  |  |
| cg14631576 | 0.56 | 0.75 | -0.19 | 1.09E-03 | 9:95140430 | *CENPP* | Body |
| cg21217540 | 0.50 | 0.69 | -0.19 | 3.23E-10 | 1:110752307 |  |  |
| cg18459618 | 0.10 | 0.29 | -0.18 | 5.48E-12 | 17:29297478 | *RNF135;RNF135* | TSS1500;TSS1500 |
| cg23899408 | 0.25 | 0.44 | -0.18 | 1.39E-03 | 19:12877188 | *HOOK2;HOOK2* | Body;Body |
| cg25834613 | 0.34 | 0.52 | -0.18 | 1.27E-04 | 7:1915315 | *MAD1L1;MAD1L1;MAD1L1* | Body;Body;Body |
| cg03433313 | 0.63 | 0.81 | -0.18 | 8.93E-04 | 16:819064 | *MIR662* | TSS1500 |
| cg17425069 | 0.65 | 0.83 | -0.18 | 4.95E-06 | 7:149964288 | *ACTR3C;ACTR3C* | Body;Body |
| cg14317384 | 0.54 | 0.72 | -0.18 | 8.84E-03 | 8:216788 |  |  |
| cg14906510 | 0.66 | 0.84 | -0.18 | 8.18E-04 | 12:7781169 |  |  |
| cg24883219 | 0.44 | 0.62 | -0.18 | 4.93E-02 | 18:14457820 |  |  |
| cg26146732 | 0.57 | 0.74 | -0.17 | 2.31E-05 | 14:70690454 |  |  |
| cg17401179 | 0.32 | 0.50 | -0.17 | 1.37E-05 | 5:400517 | *AHRR* | Body |
| cg20923605 | 0.46 | 0.63 | -0.17 | 7.71E-06 | 13:112978683 |  |  |
| cg01052274 | 0.72 | 0.89 | -0.17 | 4.75E-03 | 16:87450089 | *ZCCHC14* | Body |
| cg09419670 | 0.16 | 0.33 | -0.17 | 1.23E-03 | 9:123605666 | *PSMD5;LOC253039* | TSS1500;Body |
| cg01996567 | 0.42 | 0.59 | -0.17 | 2.01E-03 | 7:149484880 | *SSPO* | Body |
| cg07970752 | 0.70 | 0.87 | -0.17 | 3.61E-05 | 16:817463 | *MSLN;MSLN* | Body;Body |
| cg18872420 | 0.66 | 0.83 | -0.17 | 2.95E-06 | 14:78023429 | *SPTLC2* | Body |
| cg25303761 | 0.37 | 0.54 | -0.17 | 8.08E-03 | 1:31256028 |  |  |
| cg00471190 | 0.19 | 0.36 | -0.17 | 3.40E-08 | 16:3115809 | *IL32;IL32;IL32;IL32;IL32;IL32;IL32;IL32;IL32* | 5'UTR;1stExon;5'UTR;5'UTR;5'UTR;5'UTR;5'UTR;5'UTR;5'UTR |
| cg08884363 | 0.52 | 0.69 | -0.17 | 6.34E-07 | 17:37377983 | *STAC2* | Body |
| cg20803293 | 0.20 | 0.36 | -0.17 | 1.35E-02 | 1:110254709 | *GSTM5* | TSS200 |
| cg26832639 | 0.73 | 0.89 | -0.17 | 5.02E-03 | 19:51457480 | *KLK5;KLK5;KLK5* | TSS1500;TSS1500;TSS1500 |
| cg13724170 | 0.62 | 0.79 | -0.17 | 9.35E-06 | 3:12713226 |  |  |
| cg02197629 | 0.26 | 0.42 | -0.16 | 8.84E-05 | 21:15352848 | *C21orf81* | TSS200 |
| cg10295955 | 0.35 | 0.52 | -0.16 | 4.80E-03 | 4:187884368 |  |  |
| cg06957310 | 0.65 | 0.81 | -0.16 | 7.70E-12 | 17:29283870 | *ADAP2* | Body |
| cg22349229 | 0.65 | 0.82 | -0.16 | 9.25E-04 | 12:96115707 | *NTN4* | Body |
| cg18924562 | 0.18 | 0.34 | -0.16 | 7.13E-05 | 12:10242785 | *CLEC1A* | Body |
| cg25593510 | 0.37 | 0.53 | -0.16 | 2.49E-03 | 1:110254662 | *GSTM5* | TSS1500 |
| cg02939781 | 0.44 | 0.60 | -0.16 | 6.94E-04 | 3:183208857 | *KLHL6* | 3'UTR |
| cg14156792 | 0.37 | 0.53 | -0.16 | 4.48E-07 | 1:870958 | *SAMD11* | Body |
| cg00568384 | 0.48 | 0.64 | -0.16 | 1.11E-05 | 2:173539262 |  |  |
| cg02978505 | 0.34 | 0.50 | -0.16 | 1.24E-04 | 16:85292322 |  |  |
| cg24895155 | 0.35 | 0.51 | -0.16 | 7.08E-07 | 8:127837199 |  |  |
| cg02022375 | 0.73 | 0.89 | -0.16 | 7.03E-05 | 17:39197721 | *KRTAP1-1* | TSS200 |
| cg17996830 | 0.59 | 0.75 | -0.16 | 6.13E-09 | 11:78616440 | *ODZ4* | Body |
| cg22343728 | 0.52 | 0.67 | -0.16 | 1.45E-04 | 22:46287028 |  |  |
| cg03878190 | 0.31 | 0.46 | -0.16 | 2.76E-07 | 2:220047358 | *FAM134A* | 3'UTR |
| cg25399239 | 0.17 | 0.32 | -0.16 | 4.56E-04 | 6:139013092 |  |  |
| cg12784228 | 0.72 | 0.88 | -0.15 | 9.67E-07 | 7:140825201 |  |  |
| cg15506703 | 0.58 | 0.73 | -0.15 | 1.93E-02 | 2:242844601 |  |  |
| cg21934190 | 0.65 | 0.80 | -0.15 | 6.67E-07 | 8:142363160 |  |  |
| cg01727431 | 0.34 | 0.49 | -0.15 | 3.06E-06 | 1:871057 | *SAMD11* | Body |
| cg11680055 | 0.16 | 0.31 | -0.15 | 3.68E-03 | 1:110230252 | *GSTM1;GSTM1* | TSS200;TSS200 |
| cg18200150 | 0.52 | 0.67 | -0.15 | 7.98E-04 | 17:30822561 | *MYO1D* | Body |
| cg27421267 | 0.48 | 0.63 | -0.15 | 4.70E-05 | 10:530836 | *DIP2C* | Body |
| cg15679892 | 0.52 | 0.67 | -0.15 | 1.02E-03 | 7:1267057 |  |  |
| cg03278514 | 0.41 | 0.55 | -0.15 | 4.44E-03 | 15:70779346 |  |  |
| cg20291162 | 0.48 | 0.62 | -0.15 | 6.20E-08 | 17:40259547 | *DHX58* | Body |
| cg02228675 | 0.12 | 0.27 | -0.15 | 5.45E-06 | 17:40259724 | *DHX58* | Body |
| cg01874867 | 0.42 | 0.57 | -0.15 | 7.27E-04 | 7:94954059 | *PON1* | TSS200 |
| cg17165241 | 0.58 | 0.73 | -0.15 | 6.41E-03 | 11:400385 | *PKP3* | Body |
| cg26751588 | 0.64 | 0.78 | -0.15 | 1.96E-03 | 17:14109671 | *COX10* | Body |
| cg20126656 | 0.60 | 0.75 | -0.15 | 2.25E-05 | 17:76801712 | *USP36* | Body |
| cg09035930 | 0.63 | 0.78 | -0.15 | 8.41E-04 | 12:129282057 | *SLC15A4* | Body |
| cg11358405 | 0.31 | 0.45 | -0.15 | 5.01E-05 | 15:91382972 |  |  |
| cg09404119 | 0.45 | 0.60 | -0.15 | 4.79E-04 | 5:565486 |  |  |
| cg04480106 | 0.71 | 0.86 | -0.15 | 1.16E-03 | 5:72934606 | *RGNEF* | 5'UTR |
| cg08979191 | 0.73 | 0.88 | -0.14 | 1.75E-06 | 5:132113734 | *SEPT8;SEPT8;SEPT8;SEPT8* | TSS200;TSS1500;TSS1500;TSS1500 |
| cg00071950 | 0.12 | 0.26 | -0.14 | 5.36E-05 | 4:10020882 | *SLC2A9;SLC2A9* | Body;Body |
| cg23666844 | 0.45 | 0.60 | -0.14 | 5.71E-03 | 7:151137882 | *CRYGN* | TSS1500 |
| cg05293897 | 0.33 | 0.48 | -0.14 | 1.29E-05 | 2:226913713 |  |  |
| cg07157030 | 0.51 | 0.65 | -0.14 | 3.79E-02 | 14:63671356 | *RHOJ;RHOJ* | 5'UTR;1stExon |
| cg22964396 | 0.27 | 0.41 | -0.14 | 4.95E-06 | 11:71524540 |  |  |
| cg17330251 | 0.48 | 0.62 | -0.14 | 8.10E-04 | 7:94953956 | *PON1* | TSS200 |
| cg19980771 | 0.24 | 0.38 | -0.14 | 1.74E-02 | 6:110798022 | *SLC22A16* | TSS200 |
| cg06223162 | 0.42 | 0.56 | -0.14 | 4.34E-02 | 1:101003688 | *GPR88* | TSS200 |
| cg14021170 | 0.41 | 0.55 | -0.14 | 1.08E-05 | 9:826657 |  |  |
| cg24838063 | 0.69 | 0.83 | -0.14 | 2.88E-03 | 12:130822603 | *PIWIL1* | TSS200 |
| cg20135711 | 0.64 | 0.78 | -0.14 | 2.03E-05 | 12:53592259 | *ITGB7* | Body |
| cg21717724 | 0.62 | 0.76 | -0.14 | 3.39E-03 | 9:123604514 | *PSMD5;LOC253039* | Body;TSS1500 |
| cg20846728 | 0.61 | 0.75 | -0.14 | 1.60E-03 | 2:1843184 | *MYT1L* | Body |
| cg08322580 | 0.60 | 0.74 | -0.14 | 1.12E-05 | 14:70690538 |  |  |
| cg07010633 | 0.45 | 0.59 | -0.14 | 1.47E-08 | 17:73824396 | *UNC13D* | Body |
| cg14157578 | 0.42 | 0.55 | -0.14 | 2.49E-03 | 9:110764967 |  |  |
| cg06630425 | 0.79 | 0.92 | -0.14 | 3.13E-05 | 16:818451 | *MSLN;MSLN* | Body;Body |
| cg14409029 | 0.47 | 0.60 | -0.14 | 3.34E-03 | 11:19138243 | *ZDHHC13;ZDHHC13* | TSS1500;TSS1500 |
| cg08477687 | 0.36 | 0.50 | -0.14 | 2.41E-02 | 1:566570 | *MIR1977* | TSS1500 |
| cg21789181 | 0.17 | 0.30 | -0.14 | 1.03E-07 | 3:98376427 |  |  |
| cg14161159 | 0.61 | 0.74 | -0.14 | 2.95E-02 | 2:132481826 | *C2orf27A* | 5'UTR |
| cg16866567 | 0.06 | 0.19 | -0.14 | 2.46E-03 | 8:38759261 | *PLEKHA2* | 5'UTR |
| cg03116837 | 0.49 | 0.63 | -0.14 | 2.05E-03 | 8:216703 |  |  |
| cg22725940 | 0.44 | 0.57 | -0.14 | 3.21E-05 | X:119028450 | *AKAP14;AKAP14;AKAP14* | TSS1500;TSS1500;TSS1500 |
| cg14017402 | 0.33 | 0.47 | -0.14 | 1.93E-02 | 2:86225602 |  |  |
| cg21233003 | 0.47 | 0.61 | -0.14 | 1.43E-06 | 9:140057464 | *GRIN1;GRIN1;GRIN1* | Body;Body;Body |
| cg08978665 | 0.26 | 0.40 | -0.13 | 4.03E-05 | 16:3115707 | *IL32;IL32;IL32;IL32;IL32;IL32;IL32;IL32;IL32* | 5'UTR;5'UTR;1stExon;TSS200;5'UTR;5'UTR;5'UTR;5'UTR;5'UTR |
| cg22968622 | 0.04 | 0.18 | -0.13 | 2.82E-02 | 17:43663579 |  |  |
| cg17658113 | 0.64 | 0.77 | -0.13 | 4.18E-02 | 11:1413282 | *BRSK2* | Body |
| cg00219169 | 0.44 | 0.57 | -0.13 | 6.11E-04 | 10:530951 | *DIP2C* | Body |
| cg15844419 | 0.66 | 0.80 | -0.13 | 3.04E-07 | 21:46850665 | *COL18A1* | Body |
| cg22676075 | 0.51 | 0.64 | -0.13 | 2.84E-02 | 6:135203613 |  |  |
| cg12080831 | 0.33 | 0.46 | -0.13 | 3.14E-09 | 2:121697428 | *GLI2* | Body |
| cg23206463 | 0.62 | 0.76 | -0.13 | 3.09E-05 | 5:561769 |  |  |
| cg16874583 | 0.71 | 0.84 | -0.13 | 2.86E-03 | 12:7781004 |  |  |
| cg26530341 | 0.66 | 0.80 | -0.13 | 7.44E-12 | 8:23083353 | *TNFRSF10A* | TSS1500 |
| cg14943796 | 0.47 | 0.61 | -0.13 | 4.88E-06 | 17:79377272 | *BAHCC1* | Body |
| cg18681837 | 0.35 | 0.48 | -0.13 | 8.05E-05 | 21:15352213 | *C21orf81* | Body |
| cg12319143 | 0.58 | 0.71 | -0.13 | 4.09E-02 | 10:134726771 |  |  |
| cg22816218 | 0.70 | 0.83 | -0.13 | 3.77E-11 | 6:3091462 | *RIPK1* | Body |
| cg18771300 | 0.45 | 0.58 | -0.13 | 4.50E-02 | 14:63671737 | *RHOJ* | 1stExon |
| cg03457142 | 0.56 | 0.69 | -0.13 | 3.18E-07 | 3:71804859 | *EIF4E3;EIF4E3* | TSS1500;TSS1500 |
| cg11266682 | 0.40 | 0.52 | -0.13 | 1.62E-04 | 4:10021025 | *SLC2A9;SLC2A9* | Body;Body |
| cg21210642 | 0.56 | 0.68 | -0.13 | 7.01E-03 | 9:100881995 | *TRIM14;TRIM14;TRIM14;TRIM14* | TSS1500;TSS1500;TSS1500;TSS1500 |
| cg06193043 | 0.60 | 0.73 | -0.13 | 2.30E-06 | 1:11908199 | *NPPA* | TSS1500 |
| cg08644463 | 0.55 | 0.68 | -0.13 | 6.31E-04 | 1:110106962 | *GNAI3* | Body |
| cg14789911 | 0.57 | 0.70 | -0.13 | 5.06E-03 | 21:47582049 | *C21orf56;C21orf56* | Body;Body |
| cg25114033 | 0.65 | 0.78 | -0.13 | 2.53E-04 | X:49060744 |  |  |
| cg19518672 | 0.43 | 0.55 | -0.13 | 1.16E-07 | 5:132113620 | *SEPT8;SEPT8;SEPT8;SEPT8* | TSS200;TSS1500;TSS1500;TSS1500 |
| cg11766577 | 0.50 | 0.63 | -0.13 | 3.23E-02 | 21:47581405 | *C21orf56;C21orf56* | Body;Body |
| cg19678392 | 0.54 | 0.66 | -0.13 | 7.95E-04 | 7:94953810 | *PON1;PON1* | 1stExon;5'UTR |
| cg02627403 | 0.51 | 0.63 | -0.13 | 8.16E-10 | 17:73823769 | *UNC13D* | 3'UTR |
| cg00590260 | 0.46 | 0.59 | -0.12 | 8.78E-04 | 10:530805 | *DIP2C* | Body |
| cg26088294 | 0.64 | 0.77 | -0.12 | 2.59E-03 | 4:132652001 |  |  |
| cg04300684 | 0.42 | 0.55 | -0.12 | 9.95E-07 | 4:1523326 |  |  |
| cg12858902 | 0.19 | 0.32 | -0.12 | 4.97E-03 | 1:110254854 | *GSTM5* | TSS200 |
| cg23100626 | 0.56 | 0.68 | -0.12 | 2.31E-04 | 2:96804247 | *ASTL* | TSS200 |
| cg02505588 | 0.72 | 0.84 | -0.12 | 2.28E-05 | 5:105753028 |  |  |
| cg14641909 | 0.60 | 0.72 | -0.12 | 1.62E-04 | 7:72320687 |  |  |
| cg05376982 | 0.39 | 0.51 | -0.12 | 6.78E-03 | 1:110254692 | *GSTM5* | TSS200 |
| cg22681495 | 0.75 | 0.87 | -0.12 | 7.79E-03 | 15:86058755 | *AKAP13;AKAP13* | Body;Body |
| cg14255062 | 0.38 | 0.50 | -0.12 | 2.08E-02 | 9:123606675 | *PSMD5;LOC253039* | TSS1500;Body |
| cg08052120 | 0.52 | 0.64 | -0.12 | 1.52E-08 | 19:52409580 | *ZNF649* | TSS1500 |
| cg17099656 | 0.11 | 0.24 | -0.12 | 1.24E-02 | 22:47135171 | *CERK* | TSS1500 |
| cg27309611 | 0.38 | 0.50 | -0.12 | 8.35E-08 | 5:132113725 | *SEPT8;SEPT8;SEPT8;SEPT8* | TSS200;TSS1500;TSS1500;TSS1500 |
| cg06046490 | 0.29 | 0.41 | -0.12 | 2.28E-05 | 11:320940 | *IFITM3* | TSS200 |
| cg18451016 | 0.57 | 0.69 | -0.12 | 3.04E-03 | 1:38461880 |  |  |
| cg14964336 | 0.11 | 0.23 | -0.12 | 3.80E-03 | 4:1523275 |  |  |
| cg24082121 | 0.46 | 0.58 | -0.12 | 7.09E-03 | 5:672872 | *TPPP* | Body |
| cg12095533 | 0.53 | 0.66 | -0.12 | 1.71E-07 | 14:105373082 |  |  |
| cg11715858 | 0.46 | 0.58 | -0.12 | 1.27E-03 | 7:1267228 |  |  |
| cg05202204 | 0.37 | 0.49 | -0.12 | 2.27E-07 | 1:38460950 |  |  |
| cg11202023 | 0.68 | 0.80 | -0.12 | 4.48E-04 | 4:73437692 |  |  |
| cg22360016 | 0.42 | 0.55 | -0.12 | 3.43E-04 | 2:105276153 |  |  |
| cg20119798 | 0.49 | 0.61 | -0.12 | 2.38E-03 | 7:94954144 | *PON1* | TSS1500 |
| cg02771260 | 0.49 | 0.61 | -0.12 | 1.69E-02 | 11:59836817 | *MS4A3;MS4A3;MS4A3* | Body;Body;Body |
| cg20966270 | 0.43 | 0.55 | -0.12 | 2.44E-06 | 1:110752047 |  |  |
| cg26942432 | 0.51 | 0.63 | -0.12 | 2.07E-03 | 15:99557464 |  |  |
| cg06468108 | 0.45 | 0.57 | -0.12 | 1.51E-04 | 2:173539091 |  |  |
| cg05122437 | 0.59 | 0.71 | -0.12 | 1.96E-03 | 13:30055092 | *MTUS2;MTUS2* | Body;Body |
| cg23958373 | 0.70 | 0.82 | -0.12 | 3.07E-02 | 8:599963 |  |  |
| cg22216157 | 0.69 | 0.81 | -0.12 | 4.44E-04 | 7:157643037 | *PTPRN2;PTPRN2;PTPRN2* | Body;Body;Body |
| cg15462411 | 0.25 | 0.37 | -0.12 | 6.88E-03 | 17:79361621 |  |  |
| cg08900409 | 0.61 | 0.73 | -0.12 | 1.27E-02 | 19:18475669 | *PGPEP1* | 3'UTR |
| cg27311590 | 0.68 | 0.80 | -0.12 | 8.77E-04 | 4:1521984 |  |  |
| cg19988367 | 0.33 | 0.45 | -0.12 | 2.84E-05 | 6:21808465 | *FLJ22536* | Body |
| cg10003262 | 0.58 | 0.70 | -0.12 | 4.06E-05 | 17:1106589 |  |  |
| cg26660312 | 0.30 | 0.42 | -0.12 | 5.49E-03 | 7:5271480 | *WIPI2;WIPI2;WIPI2;WIPI2;WIPI2* | 3'UTR;3'UTR;3'UTR;3'UTR;3'UTR |
| cg15641398 | 0.50 | 0.62 | -0.12 | 3.23E-04 | 7:1266616 |  |  |
| cg25835179 | 0.28 | 0.40 | -0.12 | 1.10E-03 | 11:67418291 | *ACY3* | TSS200 |
| cg27184903 | 0.61 | 0.73 | -0.12 | 4.80E-08 | 15:29285727 | *APBA2;APBA2* | 5'UTR;5'UTR |
| cg11438552 | 0.19 | 0.31 | -0.12 | 6.21E-04 | 22:18919803 | *PRODH* | Body |
| cg14456004 | 0.10 | 0.21 | -0.12 | 4.80E-03 | 13:21872349 |  |  |
| cg00784671 | 0.55 | 0.67 | -0.12 | 1.99E-02 | 22:46762841 | *CELSR1* | Body |
| cg16259171 | 0.66 | 0.77 | -0.12 | 5.93E-07 | 11:73660510 | *DNAJB13* | TSS1500 |
| cg13861644 | 0.59 | 0.71 | -0.12 | 3.12E-04 | 12:130822286 | *PIWIL1* | TSS1500 |
| cg26968620 | 0.39 | 0.51 | -0.12 | 1.65E-04 | X:24378204 |  |  |
| cg10950028 | 0.32 | 0.44 | -0.12 | 1.77E-02 | 1:110230633 | *GSTM1;GSTM1* | Body;Body |
| cg15969227 | 0.33 | 0.44 | -0.12 | 1.05E-03 | 6:26745279 |  |  |
| cg22798247 | 0.74 | 0.86 | -0.12 | 7.18E-03 | 6:32807372 | *TAP2;TAP2* | TSS1500;TSS1500 |
| cg00162952 | 0.59 | 0.71 | -0.12 | 2.20E-04 | 2:132057821 |  |  |
| cg18753928 | 0.32 | 0.43 | -0.12 | 1.12E-02 | 3:113234510 | *CCDC52* | TSS1500 |
| cg26776957 | 0.74 | 0.86 | -0.12 | 7.50E-07 | 11:73357183 | *PLEKHB1;PLEKHB1;PLEKHB1;PLEKHB1* | TSS200;TSS200;TSS1500;TSS1500 |
| cg06825163 | 0.50 | 0.62 | -0.12 | 1.07E-02 | 1:202172912 | *LGR6;LGR6;LGR6* | 1stExon;Body;5'UTR |
| cg02698580 | 0.67 | 0.78 | -0.12 | 2.18E-06 | 10:86016643 | *RGR;RGR;RGR* | Body;Body;Body |
| cg00794174 | 0.61 | 0.73 | -0.12 | 2.96E-02 | 12:131196417 |  |  |
| cg24131574 | 0.53 | 0.64 | -0.12 | 1.88E-04 | 5:561954 |  |  |
| cg12091331 | 0.63 | 0.74 | -0.11 | 1.20E-10 | 8:42065314 | *PLAT;PLAT* | TSS200;TSS200 |
| cg09203501 | 0.19 | 0.31 | -0.11 | 4.44E-03 | 7:5271545 | *WIPI2;WIPI2;WIPI2;WIPI2;WIPI2* | 3'UTR;3'UTR;3'UTR;3'UTR;3'UTR |
| cg03848129 | 0.33 | 0.44 | -0.11 | 3.51E-06 | 10:27541626 | *LOC387646* | TSS1500 |
| cg05082466 | 0.55 | 0.66 | -0.11 | 3.66E-02 | 6:2953123 | *SERPINB6* | Body |
| cg24135491 | 0.48 | 0.59 | -0.11 | 2.61E-05 | 17:4487099 | *SMTNL2;SMTNL2* | TSS200;TSS1500 |
| cg14641625 | 0.53 | 0.64 | -0.11 | 1.94E-02 | 5:171485294 | *STK10* | Body |
| cg09955645 | 0.44 | 0.56 | -0.11 | 2.03E-04 | 10:531098 | *DIP2C* | Body |
| cg04548204 | 0.33 | 0.44 | -0.11 | 8.92E-03 | 12:9162872 | *KLRG1* | 3'UTR |
| cg23813257 | 0.21 | 0.32 | -0.11 | 4.40E-06 | 16:3115286 | *IL32;IL32;IL32;IL32;IL32;IL32;IL32;IL32* | TSS1500;TSS200;TSS1500;TSS1500;TSS200;TSS200;TSS200;TSS1500 |
| cg17098979 | 0.58 | 0.70 | -0.11 | 6.60E-03 | 2:241562085 |  |  |
| cg27266060 | 0.17 | 0.28 | -0.11 | 2.34E-03 | 8:22091797 |  |  |
| cg14596589 | 0.39 | 0.50 | -0.11 | 7.12E-03 | 5:1107148 | *SLC12A7* | Body |
| cg18896979 | 0.68 | 0.80 | -0.11 | 1.46E-03 | 2:242844174 |  |  |
| cg03748376 | 0.07 | 0.18 | -0.11 | 3.25E-02 | 1:248100585 | *OR2L13;OR2L13* | 1stExon;5'UTR |
| cg27306467 | 0.29 | 0.40 | -0.11 | 5.76E-05 | 21:15352940 | *C21orf81* | TSS200 |
| cg24027477 | 0.70 | 0.81 | -0.11 | 1.38E-08 | 8:53883763 |  |  |
| cg03461641 | 0.73 | 0.85 | -0.11 | 5.20E-05 | 4:26454190 |  |  |
| cg19786602 | 0.45 | 0.56 | -0.11 | 2.14E-03 | 17:7966326 |  |  |
| cg21950155 | 0.55 | 0.66 | -0.11 | 4.22E-02 | 13:28555387 | *PRHOXNB* | Body |
| cg12236088 | 0.44 | 0.55 | -0.11 | 5.61E-04 | 2:105275812 |  |  |
| cg25308322 | 0.57 | 0.69 | -0.11 | 2.42E-06 | 13:86268291 |  |  |
| cg21414902 | 0.76 | 0.87 | -0.11 | 1.06E-02 | 19:2131880 | *AP3D1;AP3D1* | Body;Body |
| cg09913449 | 0.65 | 0.76 | -0.11 | 4.65E-02 | 8:42400586 | *C8orf40;C8orf40;C8orf40;C8orf40* | 5'UTR;5'UTR;5'UTR;5'UTR |
| cg01119135 | 0.70 | 0.81 | -0.11 | 4.91E-08 | 1:207205525 | *C1orf116;C1orf116* | 5'UTR;5'UTR |
| cg12580445 | 0.29 | 0.40 | -0.11 | 1.59E-02 | 5:121414634 | *LOX* | TSS1500 |
| cg09300795 | 0.69 | 0.80 | -0.11 | 3.65E-02 | 16:4042428 | *ADCY9* | Body |
| cg21123355 | 0.38 | 0.49 | -0.11 | 6.88E-07 | 9:136844644 | *VAV2;VAV2* | Body;Body |
| cg06737308 | 0.56 | 0.67 | -0.11 | 2.11E-03 | 4:185021514 | *ENPP6* | Body |
| cg23112188 | 0.35 | 0.46 | -0.11 | 1.52E-05 | 14:24563095 | *PCK2;PCK2* | TSS1500;TSS1500 |
| cg02582520 | 0.67 | 0.78 | -0.11 | 4.09E-04 | 19:1009172 | *GRIN3B* | Body |
| cg23917638 | 0.24 | 0.35 | -0.11 | 7.86E-04 | 1:567501 | *MIR1977* | TSS1500 |
| cg24425727 | 0.18 | 0.29 | -0.11 | 1.19E-03 | 6:36645648 | *CDKN1A;CDKN1A* | TSS1500;TSS1500 |
| cg12407791 | 0.49 | 0.60 | -0.11 | 7.31E-09 | 17:73824354 | *UNC13D* | Body |
| cg05256605 | 0.52 | 0.62 | -0.11 | 4.44E-02 | 5:121412184 | *LOX* | Body |
| cg19046167 | 0.78 | 0.89 | -0.11 | 2.42E-03 | 17:80928561 | *B3GNTL1* | Body |
| cg23284931 | 0.73 | 0.84 | -0.11 | 3.30E-05 | 11:13983273 | *SPON1* | TSS1500 |
| cg23027329 | 0.71 | 0.82 | -0.11 | 6.16E-04 | 10:603950 | *DIP2C* | Body |
| cg24505167 | 0.20 | 0.31 | -0.11 | 7.27E-04 | 7:1915268 | *MAD1L1;MAD1L1;MAD1L1* | Body;Body;Body |
| cg26382697 | 0.70 | 0.81 | -0.11 | 6.98E-04 | 11:2406712 | *CD81* | Body |
| cg24997886 | 0.21 | 0.31 | -0.11 | 2.29E-06 | 1:871033 | *SAMD11* | Body |
| cg08260406 | 0.13 | 0.24 | -0.11 | 2.18E-02 | 1:248100407 | *OR2L13* | TSS200 |
| cg20507276 | 0.10 | 0.21 | -0.11 | 2.35E-02 | 1:248100600 | *OR2L13;OR2L13* | 1stExon;5'UTR |
| cg04871131 | 0.60 | 0.71 | -0.11 | 2.34E-03 | 7:94954202 | *PON1* | TSS1500 |
| cg13045555 | 0.11 | 0.22 | -0.11 | 1.87E-05 | 8:105342365 |  |  |
| cg22772691 | 0.25 | 0.36 | -0.11 | 2.70E-03 | 5:1104195 | *SLC12A7* | Body |
| cg06617418 | 0.74 | 0.84 | -0.11 | 1.77E-02 | X:109662113 | *AMMECR1;RGAG1* | 5'UTR;TSS200 |
| cg02392269 | 0.79 | 0.90 | -0.11 | 7.81E-03 | 11:12491646 | *PARVA* | Body |
| cg27473997 | 0.68 | 0.79 | -0.11 | 1.14E-02 | 4:9355351 | *USP17* | TSS200 |
| cg05749792 | 0.64 | 0.75 | -0.11 | 2.49E-02 | X:69397704 | *DGAT2L6* | Body |
| cg02466830 | 0.10 | 0.21 | -0.11 | 2.05E-06 | 3:98376390 |  |  |
| cg08221350 | 0.47 | 0.57 | -0.11 | 8.04E-03 | 10:134983838 | *KNDC1* | Body |
| cg08476511 | 0.45 | 0.56 | -0.11 | 9.79E-03 | 6:168435923 | *KIF25;KIF25* | Body;Body |
| cg09847153 | 0.38 | 0.48 | -0.11 | 1.92E-03 | 1:38461540 |  |  |
| cg04781796 | 0.85 | 0.95 | -0.11 | 8.04E-03 | 1:1959747 | *GABRD* | Body |
| cg02772266 | 0.60 | 0.70 | -0.11 | 1.49E-04 | 12:4060781 |  |  |
| cg11884704 | 0.59 | 0.70 | -0.11 | 7.71E-06 | 9:130854313 | *SLC25A25;SLC25A25* | Body;1stExon |
| cg02291164 | 0.45 | 0.55 | -0.11 | 4.36E-02 | 14:59296302 |  |  |
| cg14934522 | 0.43 | 0.54 | -0.11 | 6.98E-05 | 4:77618676 | *SHROOM3* | Body |
| cg23691223 | 0.40 | 0.51 | -0.11 | 1.13E-05 | 1:57785036 | *DAB1* | 5'UTR |
| cg01295249 | 0.47 | 0.58 | -0.11 | 9.94E-05 | 2:106419144 | *NCK2;NCK2* | 5'UTR;5'UTR |
| cg15332217 | 0.37 | 0.47 | -0.11 | 2.72E-02 | 13:28555064 | *PRHOXNB* | Body |
| cg10779718 | 0.07 | 0.18 | -0.11 | 1.93E-03 | 11:117014861 | *PAFAH1B2* | TSS200 |
| cg07837187 | 0.33 | 0.43 | -0.11 | 3.74E-04 | 5:116113889 |  |  |
| cg09319828 | 0.23 | 0.34 | -0.11 | 6.57E-04 | 1:156551787 | *TTC24* | Body |
| cg08944170 | 0.10 | 0.20 | -0.10 | 3.58E-02 | 1:248100614 | *OR2L13;OR2L13* | 1stExon;5'UTR |
| cg25833922 | 0.50 | 0.61 | -0.10 | 3.81E-09 | 11:7709186 |  |  |
| cg09125779 | 0.37 | 0.47 | -0.10 | 8.22E-04 | 4:57547999 | *HOPX;HOPX;HOPX* | TSS200;TSS200;TSS200 |
| cg05499111 | 0.63 | 0.74 | -0.10 | 6.61E-03 | 15:27902080 |  |  |
| cg25831233 | 0.02 | 0.13 | -0.10 | 7.22E-03 | 7:102119529 | *POLR2J* | TSS200 |
| cg10836661 | 0.57 | 0.68 | -0.10 | 1.01E-03 | 15:29093810 |  |  |
| cg12609785 | 0.09 | 0.19 | -0.10 | 2.83E-02 | 17:43660871 |  |  |
| cg15469871 | 0.23 | 0.33 | -0.10 | 4.69E-04 | 10:27541418 | *LOC387646* | TSS200 |
| cg12932613 | 0.68 | 0.78 | -0.10 | 2.93E-05 | 15:53086630 |  |  |
| cg25975856 | 0.65 | 0.75 | -0.10 | 4.37E-02 | 3:19930780 | *EFHB* | Body |
| cg09050670 | 0.62 | 0.73 | -0.10 | 6.68E-03 | 16:1521617 | *CLCN7;CLCN7* | Body;Body |
| cg00935887 | 0.50 | 0.61 | -0.10 | 1.84E-03 | 2:242844000 |  |  |
| cg17573270 | 0.56 | 0.66 | -0.10 | 2.80E-02 | 22:42950376 | *SERHL2* | Body |
| cg26116937 | 0.40 | 0.50 | -0.10 | 3.76E-04 | 10:531152 | *DIP2C* | Body |
| cg27512510 | 0.63 | 0.73 | -0.10 | 2.58E-08 | 1:149670340 |  |  |
| cg11146691 | 0.42 | 0.52 | -0.10 | 4.33E-02 | 12:47219737 | *SLC38A4;SLC38A4;SLC38A4;SLC38A4* | 1stExon;5'UTR;1stExon;5'UTR |
| cg20282814 | 0.56 | 0.66 | -0.10 | 2.24E-02 | 1:43423072 | *SLC2A1* | Body |
| cg20768743 | 0.10 | 0.20 | -0.10 | 1.02E-08 | 18:67624846 | *CD226* | TSS1500 |
| cg16532282 | 0.66 | 0.77 | -0.10 | 4.15E-04 | 1:226271799 |  |  |
| cg06486129 | 0.55 | 0.65 | -0.10 | 4.28E-04 | 21:45573410 |  |  |
| cg27006947 | 0.40 | 0.51 | -0.10 | 2.01E-03 | 7:63924280 |  |  |
| cg01907837 | 0.65 | 0.75 | -0.10 | 3.20E-05 | 6:37321337 | *RNF8;RNF8* | TSS1500;TSS1500 |
| cg27509823 | 0.72 | 0.82 | -0.10 | 2.36E-06 | 1:173930183 | *RC3H1* | Body |
| cg23108931 | 0.12 | 0.22 | -0.10 | 2.31E-05 | 8:105342351 |  |  |
| cg19346084 | 0.71 | 0.81 | -0.10 | 7.22E-03 | 22:50496499 |  |  |
| cg23719124 | 0.33 | 0.43 | -0.10 | 1.28E-02 | 1:110254919 | *GSTM5;GSTM5* | 1stExon;5'UTR |
| cg18171855 | 0.62 | 0.72 | -0.10 | 1.32E-04 | 10:2543474 |  |  |
| cg09522479 | 0.58 | 0.68 | -0.10 | 1.48E-03 | 10:604032 | *DIP2C* | Body |
| cg01290755 | 0.26 | 0.36 | -0.10 | 1.55E-02 | 12:129554587 |  |  |
| cg18190219 | 0.61 | 0.72 | -0.10 | 6.38E-03 | 22:46762943 | *CELSR1* | Body |
| cg05542681 | 0.16 | 0.26 | -0.10 | 1.13E-02 | 16:744328 | *FBXL16* | Body |
| cg01489256 | 0.80 | 0.70 | 0.10 | 4.41E-06 | 8:11204017 | *TDH* | Body |
| cg10836733 | 0.25 | 0.15 | 0.10 | 4.50E-05 | 12:7793353 |  |  |
| cg19389372 | 0.57 | 0.47 | 0.10 | 2.79E-05 | 19:36485356 | *SDHAF1* | TSS1500 |
| cg04373760 | 0.72 | 0.62 | 0.10 | 3.23E-02 | 16:53404718 |  |  |
| cg15288329 | 0.52 | 0.42 | 0.10 | 3.30E-03 | 6:170268691 |  |  |
| cg08858540 | 0.81 | 0.71 | 0.10 | 1.87E-05 | 5:346238 | *AHRR* | Body |
| cg08739696 | 0.65 | 0.55 | 0.10 | 2.33E-08 | 16:854168 | *PRR25* | TSS1500 |
| cg07125469 | 0.65 | 0.55 | 0.10 | 6.08E-05 | 6:125623573 | *HDDC2* | TSS1500 |
| cg21285198 | 0.68 | 0.58 | 0.10 | 2.18E-06 | 1:1360970 | *TMEM88B* | TSS1500 |
| cg22812413 | 0.53 | 0.43 | 0.10 | 2.42E-08 | 15:81391742 |  |  |
| cg09978860 | 0.45 | 0.35 | 0.10 | 6.45E-03 | 4:56023921 |  |  |
| cg11336860 | 0.41 | 0.31 | 0.10 | 3.20E-05 | 13:112575966 |  |  |
| cg01768082 | 0.70 | 0.60 | 0.10 | 1.44E-06 | 1:946875 |  |  |
| cg07189381 | 0.39 | 0.28 | 0.10 | 4.83E-05 | 19:55660514 | *TNNT1;TNNT1;TNNT1* | 5'UTR;5'UTR;5'UTR |
| cg24768135 | 0.60 | 0.50 | 0.10 | 1.65E-05 | 17:4805392 | *CHRNE;C17orf107* | Body;3'UTR |
| cg18649823 | 0.33 | 0.23 | 0.10 | 4.86E-05 | 19:55660591 | *TNNT1;TNNT1;TNNT1;TNNT1;TNNT1;TNNT1* | 1stExon;1stExon;5'UTR;5'UTR;1stExon;5'UTR |
| cg12301347 | 0.44 | 0.34 | 0.10 | 1.08E-04 | 22:46285638 |  |  |
| cg10365886 | 0.74 | 0.64 | 0.10 | 3.34E-02 | 6:32063874 | *TNXB* | Body |
| cg24699670 | 0.30 | 0.20 | 0.10 | 5.91E-04 | 19:55660614 | *TNNT1;TNNT1;TNNT1* | TSS200;TSS200;TSS200 |
| cg21964928 | 0.61 | 0.51 | 0.10 | 7.81E-03 | 5:153872958 |  |  |
| cg02710296 | 0.43 | 0.32 | 0.10 | 1.42E-02 | 1:182921851 | *C1orf14* | Body |
| cg04459086 | 0.53 | 0.43 | 0.10 | 7.85E-06 | 3:45429742 | *LARS2* | TSS1500 |
| cg26280727 | 0.81 | 0.71 | 0.10 | 2.29E-04 | 19:49000998 | *LMTK3* | Body |
| cg01824063 | 0.57 | 0.47 | 0.10 | 4.69E-06 | 11:71276654 | *KRTAP5-10* | 1stExon |
| cg00436969 | 0.66 | 0.55 | 0.10 | 1.79E-02 | 2:220197716 | *RESP18* | Body |
| cg24596472 | 0.36 | 0.26 | 0.10 | 9.18E-06 | 8:144417263 | *TOP1MT* | TSS1500 |
| cg02446475 | 0.54 | 0.44 | 0.10 | 7.40E-03 | 6:31275807 |  |  |
| cg22261987 | 0.78 | 0.67 | 0.10 | 3.30E-06 | 8:256888 |  |  |
| cg08355045 | 0.85 | 0.75 | 0.10 | 1.66E-04 | 6:80787529 |  |  |
| cg10099207 | 0.80 | 0.70 | 0.10 | 3.23E-02 | 15:39544144 | *C15orf54* | 5'UTR |
| cg18756179 | 0.20 | 0.09 | 0.10 | 2.24E-04 | 19:50554510 | *FLJ26850* | Body |
| cg02750262 | 0.51 | 0.40 | 0.10 | 1.26E-02 | 18:72916776 | *ZADH2* | Body |
| cg08284263 | 0.59 | 0.48 | 0.10 | 7.80E-04 | 10:92958627 |  |  |
| cg06975067 | 0.46 | 0.36 | 0.10 | 2.80E-02 | 13:20692525 |  |  |
| cg27387193 | 0.56 | 0.46 | 0.10 | 1.06E-02 | 6:32064032 | *TNXB* | Body |
| cg07123701 | 0.38 | 0.28 | 0.10 | 6.11E-06 | 4:56024434 |  |  |
| cg15576492 | 0.24 | 0.14 | 0.10 | 1.77E-02 | 1:1015447 |  |  |
| cg25513659 | 0.38 | 0.28 | 0.10 | 1.91E-05 | 17:183931 | *RPH3AL* | 5'UTR |
| cg02243278 | 0.83 | 0.72 | 0.10 | 2.38E-03 | 8:2076984 | *MYOM2* | Body |
| cg06862049 | 0.72 | 0.62 | 0.10 | 5.89E-04 | 19:49001890 | *LMTK3* | Body |
| cg15085883 | 0.67 | 0.57 | 0.10 | 1.22E-05 | 12:68848994 |  |  |
| cg14708514 | 0.35 | 0.25 | 0.10 | 3.54E-04 | 8:141609338 | *EIF2C2;EIF2C2* | Body;Body |
| cg18705808 | 0.70 | 0.59 | 0.10 | 1.50E-02 | 7:1588284 | *TMEM184A* | Body |
| cg06811094 | 0.55 | 0.45 | 0.10 | 1.19E-03 | 1:200272112 |  |  |
| cg09729012 | 0.35 | 0.25 | 0.10 | 1.28E-03 | 3:72395774 |  |  |
| cg00900933 | 0.59 | 0.49 | 0.10 | 2.56E-12 | 17:37322028 | *ARL5C;ARL5C* | 1stExon;5'UTR |
| cg24055178 | 0.62 | 0.52 | 0.10 | 1.45E-08 | 10:134263487 |  |  |
| cg19743168 | 0.44 | 0.33 | 0.10 | 4.97E-03 | 1:23544995 |  |  |
| cg04488758 | 0.48 | 0.38 | 0.10 | 3.52E-02 | 12:95945366 | *USP44* | TSS200 |
| cg22217449 | 0.72 | 0.61 | 0.10 | 2.36E-06 | 21:47845788 | *PCNT* | Body |
| cg06526721 | 0.52 | 0.42 | 0.11 | 2.10E-08 | 5:119799384 | *PRR16* | TSS1500 |
| cg21351392 | 0.80 | 0.70 | 0.11 | 2.64E-02 | 6:161607487 | *AGPAT4* | Body |
| cg16512390 | 0.68 | 0.58 | 0.11 | 1.64E-02 | 1:228756714 |  |  |
| cg04633141 | 0.95 | 0.85 | 0.11 | 3.28E-03 | 7:1588319 | *TMEM184A* | Body |
| cg07502417 | 0.49 | 0.38 | 0.11 | 5.19E-06 | 1:230849801 | *AGT* | 5'UTR |
| cg27429195 | 0.39 | 0.28 | 0.11 | 1.98E-02 | 10:1602108 | *ADARB2* | Body |
| cg10541466 | 0.71 | 0.61 | 0.11 | 8.14E-05 | 1:113425263 |  |  |
| cg11945929 | 0.47 | 0.37 | 0.11 | 4.24E-04 | 7:158750384 |  |  |
| cg09274344 | 0.52 | 0.41 | 0.11 | 9.70E-06 | 16:1078689 |  |  |
| cg13935437 | 0.30 | 0.20 | 0.11 | 1.17E-02 | 1:19600673 | *AKR7L;AKR7L* | TSS200;TSS200 |
| cg00497905 | 0.54 | 0.43 | 0.11 | 2.87E-06 | 11:76903183 | *MYO7A;MYO7A* | Body;Body |
| cg13469425 | 0.83 | 0.72 | 0.11 | 2.69E-02 | 4:48175353 | *TEC* | Body |
| cg27258561 | 0.62 | 0.51 | 0.11 | 1.84E-03 | 6:31275767 |  |  |
| cg02601489 | 0.59 | 0.49 | 0.11 | 5.93E-06 | 8:11203954 | *TDH* | Body |
| cg16777618 | 0.61 | 0.51 | 0.11 | 4.90E-03 | 11:128694184 |  |  |
| cg00813378 | 0.44 | 0.33 | 0.11 | 6.91E-04 | 1:1475209 | *C1orf70* | Body |
| cg14963928 | 0.77 | 0.66 | 0.11 | 4.31E-06 | 1:40392088 |  |  |
| cg23651889 | 0.51 | 0.40 | 0.11 | 9.42E-03 | 3:188115336 | *LPP;LPP;LPP* | 5'UTR;5'UTR;5'UTR |
| cg12908607 | 0.33 | 0.22 | 0.11 | 7.51E-07 | 1:44402522 | *ARTN;ARTN;ARTN;ARTN;ARTN* | 3'UTR;3'UTR;3'UTR;3'UTR;3'UTR |
| cg05776630 | 0.41 | 0.30 | 0.11 | 2.67E-02 | 1:20015027 | *TMCO4* | Body |
| cg03355213 | 0.56 | 0.45 | 0.11 | 8.55E-03 | 20:32398230 | *CHMP4B* | TSS1500 |
| cg14133635 | 0.31 | 0.20 | 0.11 | 7.57E-03 | 9:33749416 | *PRSS3* | TSS1500 |
| cg26891211 | 0.81 | 0.70 | 0.11 | 4.78E-05 | 16:17931006 |  |  |
| cg14270590 | 0.38 | 0.28 | 0.11 | 9.25E-04 | 14:105499706 |  |  |
| cg03718241 | 0.19 | 0.08 | 0.11 | 9.32E-08 | 6:3623988 |  |  |
| cg10079374 | 0.56 | 0.45 | 0.11 | 5.39E-04 | 7:158750417 |  |  |
| cg03066788 | 0.27 | 0.16 | 0.11 | 4.15E-06 | 8:28175285 | *PNOC* | 5'UTR |
| cg00315563 | 0.31 | 0.20 | 0.11 | 1.59E-03 | 3:182243039 |  |  |
| cg02527881 | 0.76 | 0.65 | 0.11 | 6.82E-05 | 3:46936655 | *PTH1R* | Body |
| cg08703522 | 0.55 | 0.44 | 0.11 | 7.85E-03 | 17:44898785 |  |  |
| cg25481157 | 0.53 | 0.42 | 0.11 | 2.10E-02 | 15:66947564 |  |  |
| cg27279301 | 0.65 | 0.54 | 0.11 | 4.00E-03 | 14:105945287 | *CRIP2* | Body |
| cg22125112 | 0.43 | 0.32 | 0.11 | 2.19E-03 | 3:69402811 | *FRMD4B* | Body |
| cg17831117 | 0.71 | 0.60 | 0.11 | 6.38E-03 | 17:18305305 |  |  |
| cg06419659 | 0.55 | 0.44 | 0.11 | 1.88E-02 | 1:153600132 | *S100A13;S100A13;S100A13;S100A1;S100A13;S100A13* | TSS1500;5'UTR;TSS200;TSS1500;5'UTR;TSS1500 |
| cg14384748 | 0.39 | 0.28 | 0.11 | 1.45E-02 | 12:132859951 | *GALNT9* | Body |
| cg14064268 | 0.54 | 0.43 | 0.11 | 9.58E-03 | 11:1463554 | *BRSK2* | Body |
| cg05991492 | 0.47 | 0.36 | 0.11 | 9.57E-03 | 16:3988700 |  |  |
| cg14356919 | 0.30 | 0.19 | 0.11 | 1.24E-02 | 21:46890997 | *COL18A1;COL18A1;COL18A1* | Body;Body;Body |
| cg20784950 | 0.29 | 0.18 | 0.11 | 8.75E-03 | 8:145002522 | *PLEC1;PLEC1;PLEC1;PLEC1;PLEC1;PLEC1;PLEC1;PLEC1* | Body;Body;Body;Body;Body;Body;Body;Body |
| cg08887025 | 0.67 | 0.56 | 0.11 | 3.50E-02 | 1:246415176 | *SMYD3;SMYD3* | Body;Body |
| cg12856392 | 0.64 | 0.53 | 0.11 | 5.89E-04 | 7:64126140 | *ZNF107;ZNF107* | TSS1500;TSS1500 |
| cg17158941 | 0.36 | 0.25 | 0.11 | 1.66E-04 | 7:1073255 | *C7orf50;C7orf50;C7orf50* | Body;Body;Body |
| cg14939821 | 0.62 | 0.51 | 0.11 | 2.96E-04 | 10:128947230 | *DOCK1;FAM196A* | Body;Body |
| cg07263979 | 0.76 | 0.65 | 0.11 | 8.84E-05 | 2:241585888 |  |  |
| cg16555417 | 0.53 | 0.42 | 0.11 | 3.39E-06 | 1:40598772 |  |  |
| cg22549041 | 0.52 | 0.40 | 0.11 | 1.92E-04 | 15:75019251 | *CYP1A1* | TSS1500 |
| cg18202521 | 0.23 | 0.12 | 0.11 | 9.50E-03 | 1:19600702 | *AKR7L;AKR7L* | TSS200;TSS200 |
| cg11747594 | 0.83 | 0.72 | 0.11 | 4.15E-02 | 6:29648225 |  |  |
| cg20867963 | 0.71 | 0.59 | 0.11 | 3.94E-02 | 6:3743529 | *C6orf145* | Body |
| cg22452837 | 0.34 | 0.23 | 0.11 | 3.63E-02 | 6:28664155 |  |  |
| cg27565517 | 0.53 | 0.42 | 0.11 | 3.20E-05 | 6:12292676 | *EDN1;EDN1* | Body;Body |
| cg19142026 | 0.52 | 0.41 | 0.11 | 1.54E-02 | 7:27170394 | *HOXA4;HOXA4* | 5'UTR;1stExon |
| cg00872984 | 0.54 | 0.43 | 0.11 | 2.65E-02 | 6:32063991 | *TNXB* | Body |
| cg08539872 | 0.36 | 0.25 | 0.11 | 3.21E-05 | 10:73004610 | *UNC5B* | Body |
| cg20965743 | 0.35 | 0.24 | 0.11 | 1.06E-03 | 14:104552209 | *ASPG* | Body |
| cg20313873 | 0.54 | 0.43 | 0.11 | 1.55E-04 | 10:101436820 | *ENTPD7* | Body |
| cg16194588 | 0.75 | 0.64 | 0.11 | 4.20E-05 | 19:49002477 | *LMTK3* | Body |
| cg02102533 | 0.92 | 0.81 | 0.11 | 1.18E-03 | 16:57562841 | *CCDC102A* | Body |
| cg27423627 | 0.58 | 0.47 | 0.11 | 2.66E-05 | 3:69299355 | *FRMD4B* | Body |
| cg02155558 | 0.26 | 0.14 | 0.11 | 3.35E-06 | 15:43621948 | *ADAL;LCMT2;ADAL* | TSS1500;1stExon;TSS1500 |
| cg05903289 | 0.76 | 0.65 | 0.11 | 1.73E-04 | 2:130345205 |  |  |
| cg07515196 | 0.25 | 0.13 | 0.11 | 6.38E-05 | 19:11354240 | *DOCK6* | Body |
| cg08825200 | 0.81 | 0.70 | 0.11 | 2.29E-07 | 3:178984841 | *KCNMB3;KCNMB3* | TSS200;TSS200 |
| cg12067421 | 0.41 | 0.30 | 0.11 | 6.70E-04 | 1:203246482 |  |  |
| cg13286698 | 0.57 | 0.46 | 0.11 | 3.51E-06 | 8:11203882 | *TDH* | Body |
| cg12823839 | 0.43 | 0.31 | 0.11 | 8.15E-05 | 7:155175340 |  |  |
| cg24912902 | 0.78 | 0.67 | 0.11 | 2.25E-10 | 2:121301738 |  |  |
| cg12893697 | 0.32 | 0.21 | 0.11 | 9.27E-03 | 11:970389 | *AP2A2* | Body |
| cg01256539 | 0.86 | 0.75 | 0.11 | 1.43E-09 | 5:119802022 | *PRR16* | 5'UTR |
| cg17132030 | 0.54 | 0.42 | 0.12 | 1.67E-03 | 19:17599784 | *SLC27A1* | Body |
| cg14167415 | 0.76 | 0.65 | 0.12 | 1.52E-02 | 5:77146999 |  |  |
| cg18222544 | 0.58 | 0.47 | 0.12 | 1.67E-07 | 1:114576186 |  |  |
| cg14240646 | 0.51 | 0.40 | 0.12 | 4.93E-02 | 10:27532245 | *ACBD5;ACBD5* | TSS1500;TSS1500 |
| cg26988138 | 0.34 | 0.23 | 0.12 | 1.98E-05 | 19:2639424 | *GNG7* | 5'UTR |
| cg08587775 | 0.32 | 0.21 | 0.12 | 2.48E-03 | 19:30363203 |  |  |
| cg25075347 | 0.74 | 0.63 | 0.12 | 6.57E-04 | 6:133731801 | *EYA4;EYA4;EYA4* | Body;Body;Body |
| cg01045132 | 0.38 | 0.27 | 0.12 | 1.52E-13 | 19:11649571 | *CNN1* | TSS200 |
| cg06935979 | 0.75 | 0.64 | 0.12 | 1.98E-04 | 1:232941706 | *KIAA1383* | 1stExon |
| cg24608381 | 0.51 | 0.40 | 0.12 | 6.21E-05 | 2:139538746 | *NXPH2* | TSS1500 |
| cg09093656 | 0.40 | 0.28 | 0.12 | 3.94E-05 | 17:16323481 | *TRPV2* | Body |
| cg08610426 | 0.65 | 0.54 | 0.12 | 1.52E-04 | 19:49249123 | *IZUMO1* | 5'UTR |
| cg27550663 | 0.58 | 0.47 | 0.12 | 6.28E-06 | 12:9722091 |  |  |
| cg26843498 | 0.59 | 0.47 | 0.12 | 3.48E-03 | 19:2361574 |  |  |
| cg04879348 | 0.90 | 0.78 | 0.12 | 1.21E-03 | 2:109124576 | *GCC2;GCC2* | 3'UTR;Body |
| cg07347128 | 0.50 | 0.39 | 0.12 | 2.62E-02 | 12:48336360 |  |  |
| cg24852442 | 0.55 | 0.43 | 0.12 | 1.75E-06 | 11:76903134 | *MYO7A;MYO7A* | Body;Body |
| cg07089942 | 0.58 | 0.46 | 0.12 | 1.52E-05 | 16:49666504 | *ZNF423* | Body |
| cg11685843 | 0.75 | 0.63 | 0.12 | 6.23E-03 | 4:176349171 |  |  |
| cg14940260 | 0.77 | 0.65 | 0.12 | 8.84E-06 | 6:16217882 |  |  |
| cg06905511 | 0.73 | 0.61 | 0.12 | 3.37E-04 | 5:139062493 | *CXXC5* | 3'UTR |
| cg19763428 | 0.39 | 0.28 | 0.12 | 9.30E-05 | 7:32109831 | *PDE1C* | Body |
| cg03396347 | 0.68 | 0.57 | 0.12 | 4.06E-05 | 1:1875803 |  |  |
| cg06022607 | 0.75 | 0.63 | 0.12 | 2.82E-04 | 12:133412002 |  |  |
| cg08883485 | 0.60 | 0.48 | 0.12 | 7.90E-07 | 1:201619787 | *NAV1* | Body |
| cg00951395 | 0.49 | 0.38 | 0.12 | 3.72E-03 | 1:232941775 | *KIAA1383* | 1stExon |
| cg01361499 | 0.62 | 0.50 | 0.12 | 2.48E-05 | 15:75467997 |  |  |
| cg04682580 | 0.66 | 0.54 | 0.12 | 1.76E-05 | 1:53016396 | *ZCCHC11;ZCCHC11;ZCCHC11* | 5'UTR;5'UTR;5'UTR |
| cg03363478 | 0.46 | 0.35 | 0.12 | 1.06E-02 | 6:170268808 |  |  |
| cg21786191 | 0.76 | 0.64 | 0.12 | 5.36E-05 | 18:72916012 | *ZADH2* | Body |
| cg21970626 | 0.82 | 0.70 | 0.12 | 5.85E-03 | 13:21893289 |  |  |
| cg05592035 | 0.30 | 0.18 | 0.12 | 1.94E-03 | 17:72462984 | *CD300A* | Body |
| cg19142181 | 0.58 | 0.46 | 0.12 | 4.97E-02 | 20:61591066 | *SLC17A9* | Body |
| cg07451641 | 0.22 | 0.10 | 0.12 | 1.38E-08 | 3:9029456 | *SRGAP3;SRGAP3* | Body;Body |
| cg18838701 | 0.48 | 0.36 | 0.12 | 1.37E-02 | 19:55668612 | *TNNI3* | Body |
| cg18912855 | 0.14 | 0.02 | 0.12 | 3.62E-07 | 14:105830859 | *PACS2;PACS2* | Body;Body |
| cg15488009 | 0.45 | 0.33 | 0.12 | 1.27E-06 | 19:681445 | *FSTL3* | Body |
| cg14565151 | 0.46 | 0.34 | 0.12 | 5.30E-03 | 12:95945384 | *USP44* | TSS200 |
| cg07638857 | 0.51 | 0.39 | 0.12 | 1.31E-04 | 12:99137308 | *ANKS1B;ANKS1B* | Body;Body |
| cg04134399 | 0.33 | 0.21 | 0.12 | 4.85E-05 | 1:246231142 | *SMYD3;SMYD3* | Body;Body |
| cg23224142 | 0.50 | 0.38 | 0.12 | 7.05E-09 | 8:37082537 |  |  |
| cg03818303 | 0.70 | 0.58 | 0.12 | 6.56E-07 | 3:178984858 | *KCNMB3;KCNMB3* | TSS200;TSS200 |
| cg09681977 | 0.51 | 0.39 | 0.12 | 1.78E-05 | 2:240153103 | *HDAC4* | Body |
| cg01477861 | 0.68 | 0.55 | 0.12 | 2.81E-06 | 4:77609841 | *SHROOM3* | Body |
| cg15236483 | 0.44 | 0.32 | 0.12 | 8.07E-06 | 22:46285682 |  |  |
| cg20401567 | 0.60 | 0.48 | 0.12 | 7.92E-05 | 17:46619555 |  |  |
| cg16726039 | 0.56 | 0.43 | 0.12 | 1.05E-06 | 12:133413162 |  |  |
| cg00985040 | 0.74 | 0.62 | 0.12 | 8.29E-06 | 6:37553208 |  |  |
| cg15531562 | 0.37 | 0.25 | 0.12 | 1.03E-07 | 11:65601754 | *SNX32* | Body |
| cg11376198 | 0.31 | 0.19 | 0.12 | 4.95E-03 | 1:19600730 | *AKR7L;AKR7L* | TSS200;TSS200 |
| cg05126388 | 0.82 | 0.70 | 0.12 | 1.96E-05 | 16:49670144 | *ZNF423* | Body |
| cg00225902 | 0.51 | 0.39 | 0.12 | 9.29E-04 | 3:66444122 | *LRIG1* | Body |
| cg07599786 | 0.68 | 0.55 | 0.12 | 3.75E-05 | 1:201618654 | *NAV1* | Body |
| cg24998527 | 0.64 | 0.52 | 0.12 | 5.10E-04 | 1:42611607 |  |  |
| cg20724680 | 0.77 | 0.65 | 0.12 | 3.22E-07 | 19:49000743 | *LMTK3* | Body |
| cg16569937 | 0.50 | 0.38 | 0.12 | 2.70E-08 | 5:57174377 |  |  |
| cg03482458 | 0.26 | 0.14 | 0.12 | 1.29E-03 | 22:46692211 | *CN5H6.4;GTSE1* | Body;TSS1500 |
| cg16202734 | 0.34 | 0.22 | 0.12 | 6.13E-09 | 17:37321890 | *ARL5C* | Body |
| cg09285851 | 0.82 | 0.70 | 0.12 | 1.80E-04 | 6:10099656 |  |  |
| cg24018756 | 0.58 | 0.46 | 0.12 | 2.69E-04 | 11:300286 | *IFITM5* | TSS1500 |
| cg20360395 | 0.44 | 0.31 | 0.12 | 2.56E-03 | 12:132671235 |  |  |
| cg05837990 | 0.72 | 0.60 | 0.12 | 7.69E-05 | 7:105596483 |  |  |
| cg11426075 | 0.64 | 0.51 | 0.12 | 2.27E-02 | 1:8086959 | *ERRFI1* | TSS1500 |
| cg21473142 | 0.45 | 0.32 | 0.12 | 5.62E-04 | 3:27762095 | *EOMES* | Body |
| cg10052504 | 0.65 | 0.52 | 0.12 | 5.19E-05 | 11:121243886 |  |  |
| cg07330481 | 0.85 | 0.72 | 0.12 | 4.52E-07 | 17:37322330 | *ARL5C;ARL5C* | 1stExon;5'UTR |
| cg01423393 | 0.71 | 0.58 | 0.13 | 2.15E-02 | 8:145164665 | *KIAA1875* | Body |
| cg27423959 | 0.48 | 0.35 | 0.13 | 1.62E-02 | 3:126945870 |  |  |
| cg10582608 | 0.43 | 0.31 | 0.13 | 1.25E-02 | 6:28664190 |  |  |
| cg06064525 | 0.36 | 0.24 | 0.13 | 8.55E-03 | 11:970664 | *AP2A2* | Body |
| cg04248279 | 0.73 | 0.61 | 0.13 | 3.95E-02 | 17:184833 | *RPH3AL* | 5'UTR |
| cg18955367 | 0.74 | 0.61 | 0.13 | 3.24E-05 | 19:49002338 | *LMTK3* | Body |
| cg01089249 | 0.38 | 0.25 | 0.13 | 2.27E-10 | 2:171676553 | *GAD1;GAD1* | Body;Body |
| cg07933656 | 0.58 | 0.45 | 0.13 | 1.68E-04 | 17:20445757 |  |  |
| cg05321225 | 0.79 | 0.67 | 0.13 | 1.12E-08 | 14:104552397 | *ASPG* | Body |
| cg20737388 | 0.66 | 0.54 | 0.13 | 1.85E-03 | 11:73668626 | *DNAJB13* | Body |
| cg01329005 | 0.35 | 0.22 | 0.13 | 8.23E-05 | 19:17516712 | *BST2* | TSS1500 |
| cg07179000 | 0.84 | 0.72 | 0.13 | 6.46E-03 | 15:33023586 | *GREM1* | 3'UTR |
| cg20663365 | 0.78 | 0.65 | 0.13 | 4.63E-07 | 2:127729097 |  |  |
| cg18929511 | 0.53 | 0.40 | 0.13 | 6.33E-06 | 8:17942993 | *ASAH1;ASAH1;ASAH1* | TSS1500;TSS1500;TSS1500 |
| cg12744031 | 0.27 | 0.15 | 0.13 | 6.87E-03 | 7:158751184 |  |  |
| cg16523868 | 0.35 | 0.22 | 0.13 | 3.52E-06 | 3:140974967 | *ACPL2;ACPL2* | 5'UTR;5'UTR |
| cg07524919 | 0.58 | 0.45 | 0.13 | 8.70E-03 | 6:32063901 | *TNXB* | Body |
| cg17429870 | 0.62 | 0.49 | 0.13 | 2.26E-03 | 11:1463662 | *BRSK2* | Body |
| cg05406088 | 0.35 | 0.22 | 0.13 | 1.35E-02 | 15:66947617 |  |  |
| cg05681977 | 0.30 | 0.17 | 0.13 | 1.87E-06 | 8:145638934 | *SLC39A4;SLC39A4* | Body;Body |
| cg25332918 | 0.78 | 0.66 | 0.13 | 9.90E-04 | 7:158766061 |  |  |
| cg27037937 | 0.45 | 0.32 | 0.13 | 9.07E-06 | 15:93876630 |  |  |
| cg01348922 | 0.54 | 0.41 | 0.13 | 6.28E-05 | 17:77707012 | *ENPP7* | Body |
| cg02882979 | 0.75 | 0.62 | 0.13 | 1.70E-03 | 14:101512592 | *MIR487B;MIR539* | TSS200;TSS1500 |
| cg09791621 | 0.31 | 0.18 | 0.13 | 1.40E-04 | 16:1078722 |  |  |
| cg05064044 | 0.46 | 0.33 | 0.13 | 8.09E-04 | 6:292385 | *DUSP22;DUSP22* | 1stExon;5'UTR |
| cg00278517 | 0.53 | 0.40 | 0.13 | 7.18E-04 | 6:167070616 | *RPS6KA2* | Body |
| cg24891660 | 0.39 | 0.26 | 0.13 | 2.56E-02 | 8:145003653 | *PLEC1;PLEC1;PLEC1;PLEC1;PLEC1;PLEC1;PLEC1;PLEC1* | Body;Body;Body;Body;Body;Body;Body;Body |
| cg04854451 | 0.28 | 0.15 | 0.13 | 1.01E-04 | 15:89011607 | *MRPL46;MRPS11;MRPS11* | TSS1500;Body;Body |
| cg19047660 | 0.42 | 0.29 | 0.13 | 1.63E-04 | 17:46619923 |  |  |
| cg09759458 | 0.44 | 0.31 | 0.13 | 6.48E-05 | 19:480216 |  |  |
| cg03972071 | 0.53 | 0.40 | 0.13 | 4.68E-02 | 18:72917163 | *ZADH2* | Body |
| cg12798157 | 0.25 | 0.11 | 0.13 | 2.59E-03 | 1:19600719 | *AKR7L;AKR7L* | TSS200;TSS200 |
| cg01129641 | 0.23 | 0.10 | 0.13 | 8.17E-06 | 2:241561237 |  |  |
| cg07307142 | 0.71 | 0.58 | 0.13 | 1.12E-04 | 16:82071433 | *HSD17B2* | Body |
| cg18228590 | 0.76 | 0.63 | 0.13 | 2.56E-03 | 5:77142469 |  |  |
| cg15374924 | 0.48 | 0.35 | 0.13 | 9.68E-06 | 11:126290232 |  |  |
| cg12954512 | 0.42 | 0.28 | 0.13 | 1.15E-04 | 7:158750244 |  |  |
| cg10122452 | 0.36 | 0.22 | 0.13 | 1.20E-04 | 22:24128767 | *SMARCB1;SMARCB1* | TSS1500;TSS1500 |
| cg10590925 | 0.62 | 0.49 | 0.13 | 1.04E-02 | 11:1463620 | *BRSK2* | Body |
| cg08471713 | 0.45 | 0.32 | 0.14 | 7.25E-03 | 17:41738893 | *MEOX1;MEOX1;MEOX1* | 1stExon;1stExon;5'UTR |
| cg14750778 | 0.32 | 0.18 | 0.14 | 3.59E-05 | 17:26899428 | *PIGS* | TSS1500 |
| cg10686758 | 0.38 | 0.24 | 0.14 | 6.11E-08 | 17:39279735 | *KRTAP4-12;KRTAP4-12* | 3'UTR;1stExon |
| cg09584650 | 0.49 | 0.36 | 0.14 | 3.35E-02 | 8:2002012 | *MYOM2* | Body |
| cg05313263 | 0.60 | 0.47 | 0.14 | 3.40E-05 | 14:100906689 | *WDR25;WDR25* | Body;Body |
| cg08010094 | 0.74 | 0.61 | 0.14 | 1.10E-03 | 2:139539001 | *NXPH2* | TSS1500 |
| cg19876987 | 0.70 | 0.56 | 0.14 | 2.66E-02 | 3:14258049 |  |  |
| cg21330207 | 0.51 | 0.37 | 0.14 | 6.68E-04 | 18:72916311 | *ZADH2* | Body |
| cg18440839 | 0.37 | 0.24 | 0.14 | 2.05E-03 | 8:141609380 | *EIF2C2;EIF2C2* | Body;Body |
| cg19169023 | 0.89 | 0.75 | 0.14 | 2.46E-03 | 15:41853346 | *TYRO3* | Body |
| cg04757492 | 0.37 | 0.23 | 0.14 | 1.89E-02 | 8:145003862 | *PLEC1;PLEC1;PLEC1;PLEC1;PLEC1;PLEC1;PLEC1;PLEC1* | Body;Body;Body;Body;Body;Body;Body;Body |
| cg05373962 | 0.75 | 0.61 | 0.14 | 1.27E-03 | 22:49881684 |  |  |
| cg05130482 | 0.38 | 0.25 | 0.14 | 6.00E-03 | 8:98271949 |  |  |
| cg23352942 | 0.53 | 0.39 | 0.14 | 1.95E-06 | 3:46931381 | *PTH1R* | Body |
| cg12454169 | 0.48 | 0.34 | 0.14 | 2.03E-02 | 2:30669597 | *LCLAT1;LCLAT1* | TSS1500;TSS1500 |
| cg13286116 | 0.71 | 0.57 | 0.14 | 2.80E-03 | 11:13302098 | *ARNTL;ARNTL;ARNTL* | 5'UTR;5'UTR;5'UTR |
| cg19637330 | 0.59 | 0.45 | 0.14 | 1.35E-02 | 1:19110922 |  |  |
| cg23758822 | 0.39 | 0.25 | 0.14 | 3.75E-02 | 17:41437982 |  |  |
| cg13590055 | 0.47 | 0.33 | 0.14 | 3.23E-04 | 18:77917647 | *LOC100130522;LOC100130522;PARD6G* | Body;Body;3'UTR |
| cg04865726 | 0.58 | 0.44 | 0.14 | 1.12E-07 | 1:1365911 |  |  |
| cg17221813 | 0.54 | 0.40 | 0.14 | 3.99E-03 | 20:61590823 | *SLC17A9* | Body |
| cg11281224 | 0.78 | 0.64 | 0.14 | 5.21E-03 | 4:60001000 |  |  |
| cg20141578 | 0.41 | 0.27 | 0.14 | 2.98E-03 | 12:12225262 | *BCL2L14;BCL2L14;BCL2L14* | 5'UTR;5'UTR;5'UTR |
| cg00506935 | 0.80 | 0.66 | 0.14 | 9.14E-09 | 15:89169262 | *AEN* | 5'UTR |
| cg11235426 | 0.45 | 0.31 | 0.14 | 1.14E-03 | 6:292522 | *DUSP22;DUSP22* | 1stExon;5'UTR |
| cg07332563 | 0.47 | 0.33 | 0.14 | 2.11E-04 | 6:291687 | *DUSP22* | TSS1500 |
| cg19197915 | 0.85 | 0.71 | 0.14 | 1.08E-04 | 8:2146804 |  |  |
| cg06223736 | 0.81 | 0.66 | 0.14 | 2.74E-03 | 2:240142694 | *HDAC4* | Body |
| cg01516881 | 0.41 | 0.26 | 0.15 | 7.45E-04 | 6:292596 | *DUSP22* | Body |
| cg21111256 | 0.42 | 0.28 | 0.15 | 4.07E-02 | 19:41386507 | *CYP2A7;CYP2A7* | Body;Body |
| cg24918767 | 0.47 | 0.33 | 0.15 | 2.36E-06 | 4:88297189 | *HSD17B11* | Body |
| cg25178900 | 0.70 | 0.55 | 0.15 | 1.28E-05 | 10:27546458 |  |  |
| cg17783317 | 0.46 | 0.31 | 0.15 | 6.10E-05 | 19:54567123 | *VSTM1;VSTM1* | 1stExon;5'UTR |
| cg01363734 | 0.71 | 0.56 | 0.15 | 3.10E-05 | 12:117482953 | *TESC;TESC;TESC* | Body;Body;Body |
| cg09084391 | 0.86 | 0.71 | 0.15 | 6.28E-06 | 5:346247 | *AHRR* | Body |
| cg17749961 | 0.27 | 0.12 | 0.15 | 1.70E-02 | 2:30669863 | *LCLAT1;LCLAT1* | TSS1500;TSS1500 |
| cg08917117 | 0.40 | 0.26 | 0.15 | 8.39E-04 | 10:115059021 |  |  |
| cg19843353 | 0.54 | 0.39 | 0.15 | 2.18E-10 | 5:180651585 | *TRIM41;TRIM41* | 1stExon;1stExon |
| cg20703997 | 0.44 | 0.29 | 0.15 | 3.35E-04 | 1:4087676 |  |  |
| cg19815565 | 0.46 | 0.31 | 0.15 | 1.79E-04 | 18:77917615 | *LOC100130522;LOC100130522;PARD6G* | Body;Body;3'UTR |
| cg23522194 | 0.88 | 0.73 | 0.15 | 5.19E-06 | 19:48565189 | *PLA2G4C;PLA2G4C;PLA2G4C* | Body;Body;Body |
| cg08288016 | 0.83 | 0.68 | 0.15 | 2.70E-06 | 4:187590536 | *FAT1* | Body |
| cg00313914 | 0.68 | 0.53 | 0.15 | 5.21E-05 | 1:201618901 | *NAV1* | Body |
| cg21848673 | 0.44 | 0.29 | 0.15 | 9.68E-06 | 15:93876581 |  |  |
| cg19448478 | 0.69 | 0.54 | 0.15 | 9.20E-10 | 2:121301692 |  |  |
| cg14604066 | 0.63 | 0.48 | 0.15 | 1.34E-06 | 9:139590572 |  |  |
| cg19204924 | 0.69 | 0.54 | 0.15 | 1.75E-06 | 19:20576478 | *ZNF826* | 3'UTR |
| cg20485607 | 0.87 | 0.72 | 0.15 | 3.11E-02 | 1:120217696 |  |  |
| cg12235788 | 0.32 | 0.17 | 0.15 | 2.71E-12 | 3:123476518 | *MYLK;MYLK;MYLK;MYLK* | Body;Body;Body;Body |
| cg23118561 | 0.90 | 0.75 | 0.15 | 8.81E-04 | 13:51565237 |  |  |
| cg12425861 | 0.21 | 0.06 | 0.15 | 8.15E-09 | 14:105830606 | *PACS2;PACS2* | Body;Body |
| cg16744531 | 0.52 | 0.37 | 0.15 | 1.43E-02 | 19:17905626 | *B3GNT3* | TSS1500 |
| cg19499452 | 0.18 | 0.03 | 0.15 | 6.92E-08 | 14:105830721 | *PACS2;PACS2* | Body;Body |
| cg06279296 | 0.55 | 0.39 | 0.15 | 5.01E-03 | 10:601816 | *DIP2C* | Body |
| cg22531183 | 0.19 | 0.03 | 0.15 | 8.25E-04 | 19:50554451 | *FLJ26850* | Body |
| cg04750100 | 0.61 | 0.46 | 0.15 | 8.15E-07 | 2:136595281 | *LCT* | TSS1500 |
| cg08895590 | 0.70 | 0.54 | 0.15 | 1.48E-03 | 1:91227319 |  |  |
| cg15652532 | 0.44 | 0.29 | 0.15 | 1.94E-02 | 2:30669759 | *LCLAT1;LCLAT1* | TSS1500;TSS1500 |
| cg05482050 | 0.27 | 0.12 | 0.16 | 1.38E-08 | 5:177409334 |  |  |
| cg20311846 | 0.59 | 0.43 | 0.16 | 1.75E-06 | 4:77356250 | *SHROOM3* | TSS200 |
| cg26099834 | 0.42 | 0.26 | 0.16 | 5.57E-03 | 15:66947568 |  |  |
| cg02583091 | 0.34 | 0.19 | 0.16 | 7.24E-07 | 8:145638881 | *SLC39A4;SLC39A4* | Body;Body |
| cg08742575 | 0.43 | 0.27 | 0.16 | 7.63E-03 | 21:47604166 | *C21orf56;C21orf56* | 5'UTR;5'UTR |
| cg09219688 | 0.61 | 0.46 | 0.16 | 5.67E-04 | 10:11789383 | *ECHDC3* | Body |
| cg10248985 | 0.81 | 0.65 | 0.16 | 8.16E-10 | 15:89169255 | *AEN* | 5'UTR |
| cg08479752 | 0.56 | 0.41 | 0.16 | 1.22E-03 | 19:54567279 | *VSTM1* | TSS200 |
| cg02584267 | 0.54 | 0.38 | 0.16 | 1.21E-03 | 10:131682251 | *EBF3* | Body |
| cg08687025 | 0.63 | 0.47 | 0.16 | 4.80E-08 | 2:127729123 |  |  |
| cg14780446 | 0.59 | 0.43 | 0.16 | 2.78E-06 | 19:48565104 | *PLA2G4C;PLA2G4C;PLA2G4C* | Body;Body;Body |
| cg24247231 | 0.56 | 0.40 | 0.16 | 1.35E-02 | 15:67904302 | *MAP2K5;MAP2K5* | Body;Body |
| cg07427475 | 0.46 | 0.30 | 0.16 | 1.38E-07 | 8:145008110 | *PLEC1;PLEC1;PLEC1;PLEC1;PLEC1;PLEC1;PLEC1;PLEC1* | Body;Body;Body;Body;Body;Body;Body;Body |
| cg12099423 | 0.44 | 0.28 | 0.16 | 3.87E-05 | 20:61590751 | *SLC17A9* | Body |
| cg02251754 | 0.69 | 0.53 | 0.16 | 8.74E-03 | 1:28572299 |  |  |
| cg02784823 | 0.81 | 0.65 | 0.16 | 4.40E-07 | 19:49000897 | *LMTK3* | Body |
| cg09125754 | 0.85 | 0.68 | 0.16 | 2.36E-06 | 2:130886714 | *POTEF;POTEF* | 1stExon;5'UTR |
| cg27182070 | 0.58 | 0.41 | 0.16 | 9.00E-03 | 1:28218282 | *RPA2* | 3'UTR |
| cg08164151 | 0.52 | 0.36 | 0.16 | 2.79E-02 | 12:131118432 |  |  |
| cg17222434 | 0.80 | 0.64 | 0.17 | 1.61E-07 | 1:100215737 | *FRRS1* | 5'UTR |
| cg01394167 | 0.63 | 0.47 | 0.17 | 5.72E-03 | 4:9479622 |  |  |
| cg06712767 | 0.68 | 0.51 | 0.17 | 4.85E-02 | 12:131118561 |  |  |
| cg12622242 | 0.59 | 0.42 | 0.17 | 1.98E-03 | 8:19203257 | *SH2D4A* | Body |
| cg05357209 | 0.36 | 0.19 | 0.17 | 1.06E-02 | 7:872208 | *UNC84A;UNC84A* | 5'UTR;Body |
| cg23283614 | 0.40 | 0.24 | 0.17 | 1.01E-13 | 14:60632532 | *DHRS7* | TSS1500 |
| cg18397450 | 0.28 | 0.11 | 0.17 | 6.37E-09 | 14:105830631 | *PACS2;PACS2* | Body;Body |
| cg20021790 | 0.44 | 0.28 | 0.17 | 4.10E-02 | 17:181288 | *RPH3AL* | 5'UTR |
| cg16240816 | 0.53 | 0.36 | 0.17 | 2.00E-05 | 2:65861662 |  |  |
| cg06898279 | 0.75 | 0.59 | 0.17 | 4.28E-08 | 14:60629192 | *DHRS7* | Body |
| cg21548813 | 0.47 | 0.30 | 0.17 | 7.22E-04 | 6:291882 | *DUSP22* | TSS1500 |
| cg14580085 | 0.77 | 0.60 | 0.17 | 2.36E-03 | 2:239553406 |  |  |
| cg01219135 | 0.65 | 0.48 | 0.17 | 5.65E-06 | 7:158766336 |  |  |
| cg14228592 | 0.42 | 0.25 | 0.17 | 2.74E-05 | 8:145639181 | *SLC39A4;SLC39A4* | Body;Body |
| cg06777732 | 0.45 | 0.28 | 0.17 | 1.46E-02 | 12:131118426 |  |  |
| cg12016809 | 0.41 | 0.24 | 0.18 | 7.06E-03 | 21:47604291 | *C21orf56;C21orf56;C21orf56;C21orf56* | 5'UTR;5'UTR;1stExon;1stExon |
| cg05401945 | 0.51 | 0.34 | 0.18 | 1.46E-04 | 3:56590734 | *CCDC66;CCDC66;CCDC66* | TSS1500;TSS1500;TSS1500 |
| cg25301532 | 0.69 | 0.52 | 0.18 | 1.73E-04 | 20:43378953 | *KCNK15* | Body |
| cg22822867 | 0.70 | 0.52 | 0.18 | 1.55E-03 | 8:145004144 | *PLEC1;PLEC1;PLEC1;PLEC1;PLEC1;PLEC1;PLEC1;PLEC1* | Body;Body;Body;Body;Body;Body;Body;Body |
| cg13679164 | 0.81 | 0.64 | 0.18 | 1.25E-02 | 6:161258479 |  |  |
| cg19388776 | 0.37 | 0.20 | 0.18 | 5.38E-06 | 3:71835112 | *PROK2;PROK2* | TSS1500;TSS1500 |
| cg03395511 | 0.52 | 0.34 | 0.18 | 5.66E-04 | 6:291903 | *DUSP22* | TSS200 |
| cg14573478 | 0.44 | 0.26 | 0.18 | 4.03E-04 | 3:140730858 |  |  |
| cg11510586 | 0.43 | 0.25 | 0.18 | 1.34E-04 | 9:72027409 |  |  |
| cg04922029 | 0.86 | 0.68 | 0.18 | 2.56E-19 | 1:159174728 | *DARC;DARC;DARC* | TSS1500;5'UTR;1stExon |
| cg07618780 | 0.65 | 0.47 | 0.18 | 1.40E-03 | 2:240142806 | *HDAC4* | Body |
| cg15383120 | 0.52 | 0.34 | 0.18 | 1.09E-03 | 6:291909 | *DUSP22* | TSS200 |
| cg10510935 | 0.57 | 0.39 | 0.18 | 2.48E-06 | 1:4059661 |  |  |
| cg04287574 | 0.54 | 0.36 | 0.18 | 6.10E-05 | 1:201619622 | *NAV1* | Body |
| cg21900799 | 0.66 | 0.48 | 0.18 | 1.27E-02 | 8:145004388 | *PLEC1;PLEC1;PLEC1;PLEC1;PLEC1;PLEC1;PLEC1;PLEC1* | Body;Body;Body;Body;Body;Body;Body;Body |
| cg10599438 | 0.45 | 0.27 | 0.18 | 1.93E-02 | 19:844589 | *PRTN3* | Body |
| cg09314196 | 0.51 | 0.32 | 0.18 | 2.75E-03 | 19:22816508 | *ZNF492* | TSS1500 |
| cg02779037 | 0.23 | 0.05 | 0.18 | 6.32E-09 | 7:4848683 | *RADIL* | Body |
| cg11775292 | 0.50 | 0.31 | 0.18 | 8.56E-05 | 15:33487631 |  |  |
| cg05570109 | 0.83 | 0.65 | 0.19 | 1.18E-04 | 2:39812572 |  |  |
| cg02331830 | 0.36 | 0.17 | 0.19 | 3.44E-08 | 8:145008288 | *PLEC1;PLEC1;PLEC1;PLEC1;PLEC1;PLEC1;PLEC1;PLEC1* | Body;Body;Body;Body;Body;Body;Body;Body |
| cg10296238 | 0.58 | 0.39 | 0.19 | 9.27E-03 | 21:47605174 | *C21orf56;C21orf56* | TSS1500;TSS1500 |
| cg01089319 | 0.41 | 0.22 | 0.19 | 1.65E-09 | 2:171676809 | *GAD1;GAD1* | Body;Body |
| cg00169354 | 0.66 | 0.47 | 0.19 | 2.95E-05 | 20:54922827 |  |  |
| cg13440692 | 0.38 | 0.19 | 0.19 | 4.32E-03 | 1:1186357 |  |  |
| cg19035788 | 0.49 | 0.29 | 0.19 | 5.42E-07 | 2:242881634 |  |  |
| cg20708141 | 0.50 | 0.31 | 0.20 | 4.37E-04 | 4:1606861 |  |  |
| cg05004142 | 0.88 | 0.68 | 0.20 | 7.65E-04 | 16:57562740 | *CCDC102A* | Body |
| cg26466789 | 0.38 | 0.18 | 0.20 | 4.22E-07 | 8:144437314 |  |  |
| cg07629625 | 0.59 | 0.39 | 0.20 | 3.06E-02 | 10:133961825 | *JAKMIP3* | Body |
| cg08089543 | 0.59 | 0.39 | 0.20 | 5.26E-04 | 4:1607111 |  |  |
| cg09873524 | 0.72 | 0.52 | 0.20 | 7.49E-13 | 15:23062853 | *NIPA1;NIPA1* | Body;5'UTR |
| cg22000984 | 0.48 | 0.28 | 0.20 | 9.07E-05 | 5:150226278 | *IRGM;IRGM* | 1stExon;5'UTR |
| cg13820391 | 0.60 | 0.40 | 0.20 | 8.65E-06 | 11:121835505 |  |  |
| cg14227325 | 0.54 | 0.33 | 0.21 | 1.78E-04 | 2:113190197 | *RGPD8;RGPD5* | Body;Body |
| cg06611532 | 0.80 | 0.59 | 0.21 | 7.01E-06 | 13:114900021 |  |  |
| cg26987645 | 0.63 | 0.43 | 0.21 | 1.16E-07 | 1:203320386 | *FMOD* | TSS200 |
| cg07870920 | 0.82 | 0.60 | 0.22 | 2.44E-03 | 4:121569769 |  |  |
| cg27387030 | 0.53 | 0.31 | 0.22 | 2.57E-07 | 1:203320541 | *FMOD* | TSS1500 |
| cg14124481 | 0.68 | 0.46 | 0.22 | 2.29E-11 | 14:106094051 |  |  |
| cg02622647 | 0.57 | 0.35 | 0.22 | 3.07E-02 | 17:43578911 |  |  |
| cg14005211 | 0.51 | 0.29 | 0.22 | 1.10E-07 | 2:171676925 | *GAD1;GAD1* | Body;Body |
| cg23244022 | 0.37 | 0.14 | 0.23 | 2.09E-07 | 8:144437350 |  |  |
| cg05867499 | 0.33 | 0.10 | 0.23 | 1.20E-10 | 7:4848814 | *RADIL* | Body |
| cg12019614 | 0.50 | 0.27 | 0.23 | 4.02E-05 | 19:11353996 | *DOCK6* | Body |
| cg15948536 | 0.53 | 0.30 | 0.23 | 1.73E-06 | 4:169770092 | *PALLD;PALLD;PALLD;PALLD* | 5'UTR;Body;Body;Body |
| cg08923669 | 0.65 | 0.42 | 0.23 | 1.85E-03 | 16:420230 | *MRPL28* | 5'UTR |
| cg07086945 | 0.49 | 0.26 | 0.23 | 7.31E-12 | 6:109036145 |  |  |
| cg22123784 | 0.55 | 0.31 | 0.24 | 1.21E-07 | 8:144437507 |  |  |
| cg07420274 | 0.65 | 0.40 | 0.24 | 1.05E-12 | 2:171676306 | *GAD1;GAD1* | Body;Body |
| cg18110333 | 0.54 | 0.29 | 0.25 | 3.16E-04 | 6:292329 | *DUSP22;DUSP22* | 1stExon;5'UTR |
| cg14837792 | 0.79 | 0.53 | 0.26 | 1.66E-05 | 14:106091981 |  |  |
| cg01710670 | 0.74 | 0.47 | 0.27 | 1.58E-03 | 16:15018856 |  |  |
| cg27207756 | 0.60 | 0.33 | 0.27 | 1.10E-03 | 4:1607291 |  |  |
| cg10832239 | 0.84 | 0.56 | 0.28 | 2.71E-03 | 14:106183770 |  |  |
| cg24851651 | 0.85 | 0.48 | 0.36 | 4.78E-05 | 11:66362959 | *CCS* | Body |
| cg21028156 | 0.47 | 0.08 | 0.39 | 1.99E-08 | X:2743660 |  |  |
